# Wavelet Analysis of Respiratory Muscle sEMG Signals during the Physiological Breakpoint of Static Dry End-Expiratory Breath-Holding in Naive Apneists: A Pilot Study

**DOI:** 10.3390/s23167200

**Published:** 2023-08-16

**Authors:** Nataša Ž. Mišić, Mirko Ostojić, Saša Cvetković, Petar Miodragović, Zdravko Aničić, Anita Kovačić Popović, Đorđe Stefanović

**Affiliations:** 1 Division of Computational Bioengineering, Research & Development Institute Lola Ltd., 11030 Belgrade, Serbia; mirko.ostojic@li.rs (M.O.); sasa.cvetkovic@li.rs (S.C.); 2 Department of Physiology, Faculty of Medicine, University of Novi Sad, 21137 Novi Sad, Serbia; petar.miodragovic@gmail.com; 3 Laboratory of Methodology and Research, Faculty of Sport and Physical Education, University of Belgrade, 11030 Belgrade, Serbia; zdravko.anicic@fsfv.bg.ac.rs; 4 Department of Defectology and Clinical Psychology, Medika College for Vocational Studies in Healthcare, 11000 Belgrade, Serbia; anitakovacic987@hotmail.com; 5 Department for Basic Sports, Faculty of Sport and Physical Education, University of Belgrade, 11030 Belgrade, Serbia; prof.djordje.stefanovic@gmail.com

**Keywords:** breath-holding, hypoxia, hypercapnia, involuntary breathing movement, muscle fiber subtypes, heart rate variability, wavelet analysis, multiresolution analysis

## Abstract

The wavelet spectral characteristics of three respiratory muscle signals (scalenus (SC), parasternal intercostal (IC), and rectus abdominis (RA)) and one locomotor muscle (brachioradialis (BR)) were analyzed in the time–frequency (T-F) domain during voluntary breath-holding (BH), with a focus on the physiological breakpoint that is commonly considered the first involuntary breathing movement (IBM) that signals the end of the easy-going phase of BH. The study was performed for an end-expiratory BH physiological breaking point maneuver on twelve healthy, physically active, naive breath-holders/apneists (six professional athletes; six recreational athletes, and two individuals in the post-COVID-19 period) using surface electromyography (sEMG). We observed individual effects that were dependent on muscle oxygenation and each person’s fitness, which were consistent with the mechanism of motor unit (MU) recruitment and the transition of slow-twitch oxidative (type 1) to fast-twitch glycolytic (type 2) muscle fibers. Professional athletes had longer BH durations (BHDs) and strong hypercapnic responses regarding the expiratory RA muscle, which is activated abruptly at higher BHDs in a person-specific range below 250 Hz and is dependent on the BHD. This is in contrast with recreational athletes, who had strong hypoxic responses regarding inspiratory IC muscle, which is activated faster and gradually in the frequency range of 250–450 Hz (independent of the person and BHD). This pilot study preliminarily indicates that it is possible to noninvasively assess the physiological characteristics of skeletal muscles, especially oxygenation, and improve physical fitness tests by determining the T-F features of elevated myoelectric IC and RA activity during BH.

## 1. Introduction

The theoretical limits of human voluntary breath-holding (apnea) are defined primarily by the metabolic and mechanical limitations of cardiorespiratory physiology. The metabolic limit is the loss of consciousness due to extreme hypoxemia and hypercapnia, while the mechanical limits are the lung compression and intrathoracic vascular distension [1,2,3]. These limits are reached only by highly trained apneists (breath-holders) due to a powerful involuntary breakpoint mechanism of prolonged apnea [1], which is activated significantly below these extreme physiologic limits. The understanding of the precise mechanism and threshold for breakpoint activation is still insufficient due to complex and integrated cardiorespiratory physiology, while the persistence of sinus arrhythmia during apnea indicates the probable continuity of the central (neural) respiratory rhythm and, thus, the impossibility of its voluntary stopping [1,4]. Decades of apnea research through the design of different breath-hold (BH) maneuvers have unequivocally shown large variations in the physiological response, thus showing the continuous and robust adaptation capability of the respiratory system to changes in the internal and external environment, thereby indicating its integrative nature [1,2,5,6,7,8,9]. Moreover, the correlation of intracellular mitochondrial respiratory metabolic processes with respiration in real time, as well as changes in the membrane potential with inhalation and exhalation [10], suggest that respiration is one of the most integrated physiological processes.

Contemporary extensive, although incomplete, knowledge about the function and control of the physiological response to apnea has been acquired by studying a number of BH maneuvers under different conditions: wet/dry, normobaric/hyperbaric, static/dynamic, repetitive/nonrepetitive, end-expiratory/end-inspiratory, with/without prior hyperventilation and/or oxygenation, with/without manipulation of perception etc. BH maneuvers have also been studied for different body positions: sitting, supine, arms up, prone, and lateral, as well as on different populations: trained/naive, healthy/unhealthy, younger/older, human, animal etc. Despite these great variations in the human physiological response to apnea, which are also ascribed to the mammalian dive reflex, its critical function is oxygen conservation to maintain cerebral functioning, while its central processes are sympathetically mediated peripheral vasoconstriction connected with initial hypertension and vagally mediated bradycardia connected with the cardiac output reduction [2,5,8]. This initial increase in mean arterial pressure (MAP) coincides with a probably also sympathetically mediated spleen contraction and the ejection of fresh blood from the spleen, which allows new oxygen to enter the bloodstream and, all together, apparently hasve a beneficial effect on the apnea onset [11].

During the apneic response, it distinguishes two basic phases—the “easy-going” phase and the “struggle” phase, and their demarcation point represents the physiological breakpoint, which is commonly considered as the first involuntary breathing movement (IBM)—the powerful contractions of the respiratory muscles during which a motivated but naive apneist will break their breath hold and reinstate breathing [1,8]. The occurrence frequency and intensity of IBMs increase toward the end of apnea, which indicates that IBM has an additional beneficial impact along with other mechanisms, although its role is obscured by the fact that some elite apneists do not experience an IBM-maintained apnea (cases in which the subject, even an elite apneist, never experiences IBM during prolonged apnea) [12]. IBMs induce short-lasting increases in the MAP accompanied by positively correlated oscillations in cerebral blood volume and oxygenated hemoglobin concentration, and they are probably a consequence of chemoreceptor stimulation and consequent efferent respiratory motor output [12,13]. IBMs also coincide with splenic volume reduction and the restoration of hemodynamics, which probably facilitates the use of the last oxygen reserves before apnea cessation [11].

Understanding the physiological responses associated with apnea has the potential to provide models that illustrate cardiorespiratory interactions, as well as more integrated physiological processes, and thereby improve physical fitness tests and training [14,15,16].

In terms of improving clinical management through the (early) diagnosis of general and specific pathogenesis, various breath-holding tests are clinically used for health risk assessment and stratification, starting with breath-holding duration (BHD) for different breath-hold maneuvers as the simplest objective measure [17,18] or integrated with other data. Of particular interest is the early diagnosis of obstructive sleep apnea–hypopnea syndrome (OSAHS), which is a common sleep disorder characterized by episodes of intermittent airway obstruction during sleep with a complete (apnea) or partial (hypopnea) reduction in airflow. OSAHS leads to sleep fragmentation and intermittent hypoxia during sleep, while, in the long term, it has also been reported to be part of age-related diseases and to accelerate aging mechanisms [19], as well as other disorders and diseases [20,21]. OSAHS is still an underdiagnosed condition, but recent studies have not only shown that OSA can be identified using simple breath-holding maneuvers performed during wakefulness [22], but some research approaches suggest that it can be modeled using prolonged breath-holding [23], which highlights the importance of the reliable identification and characterization of IBMs.

Based on our previous study [24], the first aim here was to investigate the electrophysiological behavior of respiratory muscles during IBMs. Electromyography is an experimental technique that deals with developing, recording, and analyzing myoelectric signals, which are formed by physiological variations in the state of the muscle fiber membranes [25]. It is mainly used to study the neuromuscular activation of muscles within postural tasks, functional movements, work conditions, and treatment/training regimes, thus providing an established evaluation methodology for applied research, medicine (physiotherapy/rehabilitation) [26,27], and sports performance [28]. Since surface electromyography (sEMG) signals are recorded using noninvasive electrodes on the surface of the skin over the muscles, sEMG is the chosen EMG variant in this paper.

We studied three specific respiratory muscles during IBMs (for comparison, one locomotor muscle was also included), which particularly included the following determinations:

1 To find out which muscle is most appropriate for IBM detection and characterization;
2 To test the reproducibility of physiological responses of respiratory muscles;
3 To investigate an improved time–frequency (T-F) analysis method for studying respiratory muscle sEMG signals to noninvasively assess skeletal muscle oxygenation and improve physical fitness tests.

The primary goal of our experiment was to improve physical fitness tests, which naturally implied a focus on the target demographic of healthy, physically active people. Given that there is a huge difference in the duration and intensity of physical activity between professional athletes (having about 20–40 h per week of high-intensity training) and amateur athletes (having about 6–12 h per week of moderate-intensity exercise), we also aimed to determine possible differences in the physiological responses associated with apnea between these two populations. Since the aim of this pilot study was to test the framework and obtain preliminary conclusions and directions for more in-depth future research toward improving physical fitness tests, there was no target bias in terms of male versus female numbers. In addition, the COVID-19 infection led to limitations in the composition and number of participants, as well as several limitations in the design and execution of the test. To increase the statistical significance of each measurement, two consecutive measurements that were spaced sufficiently apart for recovery were collected from each participant.

Research results in the literature have demonstrated that the wavelet transform (WT) is one of the most promising methods for T-F analysis, given that it very accurately extracts features from biomedical signals. It is widely used for the analysis and classification of EEG recordings, while, for muscle sEMG signals, it was successfully used to identify muscle fatigue [29]. This paper is one of the few in which a wavelet-based multiresolution analysis has been performed for respiratory muscle sEMG signals.

Our long-term goal is to define reliable machine learning features and a robust analysis method for noninvasive apnea/hypopnea detection (similar to [30]) and to improve physical fitness tests by establishing a closer relationship between physical conditions with BHD and sEMG spectral characteristics.

To facilitate the reading of this research paper, we refer the reader to a detailed list with Abbreviations on page 28.

## 2. Database

This descriptive and exploratory study was conducted in Belgrade, Serbia. The study was performed in accordance with appropriate data protection legislation, according to the Declaration of Helsinki, and has been approved by the Research Ethics Committee at the Faculty of Sports and Physical Education in the University of Belgrade, Serbia.

### 2.1. Participants and Procedure

All participants in this study were regularly athletic/physically active non-smokers; had a fully preserved locomotor and nervous systems; had no musculoskeletal pain; no known lasting damage to the lungs, airways, and associated musculature; and were not taking any prescription medications. The professional and amateur athletes underwent regular physical evaluations with different frequencies. The presence of acute and chronic pathology in the regions and systems of research interest (musculoskeletal, neural, cardiovascular, and respiratory) was not established for the selected participants based on the anamnesis and general physical examination that was conducted prior to the experiment by a team consisting of a medical doctor, a professor, and sport therapy expert, as well as a clinical psychologist. The participants in this study received the necessary explanations and instructions beforehand and then declared in writing that they understood the objectives and procedures, as well as agreed to participate in the study.

**Figure 1:**
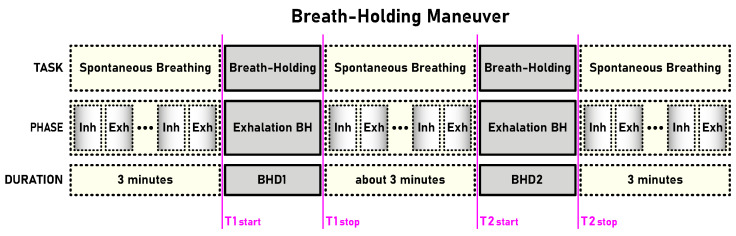
Breathing pattern of breath-holding (BH) maneuver. BH began after completion of spontaneous exhalation to functional residual capacity and ended with spontaneous inhalation to normal lung capacity. The T1start/T1stop and T2start/T2stop markers define the beginning/ending of the first and second exhalation BH, respectively (the time markers are displayed on all signal plots, whether in the time or wavelet domain, while their colors correspond to the sensor colors in Figure 2).

A schematic representation of the BH maneuver with its tasks, phases, and durations is given in Figure 1 (spontaneous breathing time frames were established based on [23,31]). Breath holding began after spontaneous exhalation to functional residual capacity (FRC), with the suggestion of refraining from preparatory hyperventilation, and ended with spontaneous inhalation. Subjects completed a shortened form of the BH maneuver in the full experimental setting as a preparatory exercise before the experimental breath holding, which consisted of the first three phases (three-minute spontaneous breathing, breath holding, and three-minute spontaneous breathing). In both cases, preparatory and experimental exercise, participants had a 5-minute rest period between the preparatory and experimental exercises.

A total of 12 healthy, physically active, naive apneists (breath holders) participated voluntarily and were divided into two equally numbered groups according to the level of sports participation—professional and amateur level. For self-assessment, participants noted their sport, age, gender, height and weight, as well as their status as a professional or amateur athlete and their main sport discipline/activity (Table 1). We performed measurements with three persons per day during the course of four days during the period from 9 a.m. to 11:30 a.m. The table additionally contains their resulting BHD during the first and second voluntary apnea defined by the BH maneuver (BHD1 and BHD2 in Figure 1).

### 2.2. Measurement and Preprocessing

**Figure 2:**
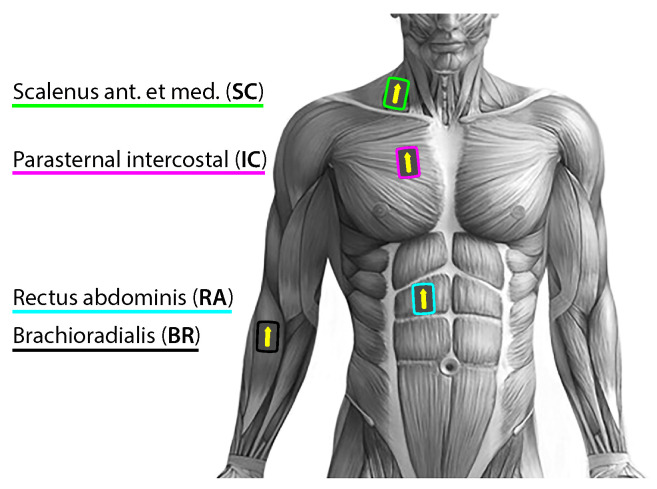
Placement of Delsys Trigno wireless sensors for unilateral right-sided body data collection of the myoelectric activity for the three respiratory muscles—the accessory inspiratory muscle scalenus anterior at medium (SC; green), the primary inspiratory muscle parasternal intercostal (IC; magenta), the accessory expiratory muscle rectus abdominis (RA; cyan), and for the locomotor muscle brachioradialis (BR; black). The colors of the sensors correspond to the colors of the time markers (T1start/T1stop and T2start/T2stop, Figure 1) on all signal plots in the time domain.

Participants performed the entire experimental test in an upright sitting position, without lumbar support, and in a quiet environment with occasional and timely notification of the remaining time until the onset of breath holding. Subjects were instructed to breathe through the nose, especially in the entry and exit phases of breath holding. They were suggested to completely relax their respiratory musculature during the early BH period, and allow respiratory contractions to develop “naturally” towards the end of breath holding. For the entire experimental setup, the choice of the right side of the body for acquisition was dictated by minimizing the interferences of cardiac activity signals, which are more pronounced on the left side of the body (see Figure 2). In addition, the subjects were instructed to avoid activating their peripheral muscles, except when indicated to mark the beginning and ending of the breath holding, which was also a sign to the operator to put and remove the nose clip. This sign was given with a slight lifting of the index finger of the left hand to further avoid activity from the right side of the body during the critical measurement phases (start and end of the BH maneuver).

The surface EMG data collection was performed using the research-orientated high-performance Trigno^tm^ Wireless EMG System (DelSys Inc., Natick, MA, USA), using four hybrid EMG/ movement Trigno sensors for the selected muscles described and depicted in Figure 2 (the recorded electromyogram signals are EMG_SC_, EMG_IC_ (electr. pos.: 2nd right IC space at the parasternal line), EMG_RA_, and EMG_BR_, respectively). The surface electromyography for the noninvasive assessment of the muscles (SENIAM: http://www.seniam.org (accessed on 14 March 2022)) project of the EU has developed important guidelines for EMG measurements in biomedical health and research areas. The sensors were applied simultaneously according to the manufacturer’s instructions and SENIAM recommendations [32,33].

Proper skin preparation was performed by shaving (hair removal), cleaning by mild scrubbing with 70% isopropyl alcohol, and then allowing the alcohol to vaporize before positioning the sensor. An arrow-shaped top of the sensor was orientated parallel to the muscle fiber direction and placed over the muscle on the mid-belly (Figure 2). Prior to sensor affixing, its precise position was determined by optimizing the signal quality during the muscle contractions using the real-time Signal Quality Tool that was included in the Delsys EMGworks^®^ Acquisition software version 4.7.8. The sensors were affixed with original Trigno Adhesive Skin Interfaces, thereby enabling a robust interface between the sensor and the skin without needing additional gels.

The sensor design as a double-differential bar sensor (four bipolar Ag/AgCl bar electrode: bar size of 5×1 mm; interelectrode spacing fixed at 10 mm; common mode rejection ratio, CMRR, of >80 dB) ensured reliability and consistency across all data collection, reduced the crosstalk signals from the adjacent muscles, and suppressed movement artifacts, as well as any common signal from distant sources such as power lines [34]. The data at the sensor level were filtered with the AC-coupling (DC-blocking) filter using a 7 Hz highpass FIR filter based on the LS algorithm and a Hamming window and with a second-order Butterworth bandpass filter between 20±5 Hz and 450±50 Hz to increase the signal-to-noise ratio, as has been similarly explained in [35]. The data were collected at a sampling frequency of 1926 Hz using a 16-bit A/D converter. In addition to hardware filtering, software filtering was performed on the raw data with a root mean square (RMS) filter. The RMS envelope calculation was performed with fully overlapped windows so that the sampling rate of the RMS would be identical to the sampling rate of the acquired data. Muscle movement artifacts were manually removed from the signal by visual inspection. All data analyses were subsequently performed in MatLab (MathWorks Inc., Natick, MA, USA).

## 3. Research Objectives and Methods

Following the set objectives of the research previously stated in the Introduction, we now present the underlying rationale and methods with the technical procedures undertaken to conduct the research process.

**Figure 3:**
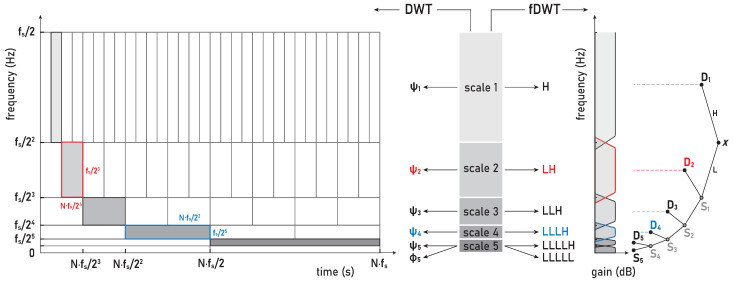
The equivalence of processing for a discrete time signal $x={\left\{x_{n}\right\}}_{n=1}^{N}$ between DWT using wavelet and scaling functions (${\left\{\Psi_{j}\right\}}_{j=1}^{5}$ and $\Phi_{5}$) and the corresponding fDWT using lowpass and highpass filters (L and H). **Left:** A perfect T-F plane tiling for DWT in dyadic (octave range) configuration, thus resulting in Heisenberg boxes with the same constant area (examples highlighted in red and blue). With successive downscaling, the wavelet is scaled by $2^{-1}$ and translated by 2. **Right:** The corresponding fDWT bandwidth division using the cascade in the form of a tree structure of halfband filters and subsampling by 2 results in filterbank with a constant relative bandwidth. Such filtering followed by sampling at the respective Nyquist frequency $f_{s}/2$ gives same wavelet and smooth coefficients (${\left\{D_{j}\right\}}_{j=1}^{5}$ and $S_{5}$) as for DWT. More detailed explanations in the main text.

Most biomedical signals are non-stationary and transient phenomena are often a research topic, as in the case of the IBM in this study. The analysis of non-stationary signals and transients demands their localization in both frequency and time. The uncertainty principle inherent in the Fourier transform, called the Heisenberg–Gabor limit, implies that localization in a time–frequency (T-F) domain is a nontrivial problem and that there is always a tradeoff between their localization. This inherent coupling between time resolution and frequency resolution in the T-F plane is represented by the minimum area of the so-called Heisenberg box [36] with a lower bound of $\frac{1}{2}$. Optimizing the T-F resolution is based on representing signals as linear superpositions of a family of functions built from translations and modulations of the generating function. This decomposition of the signals over a family of functions, called T-F atoms [36], is the basis of sparse representation theory. Depending on the choice of T-F atoms that define the basis set of the sparse transform, the decomposition can have very different properties and, thus, different T-F resolutions (Heisenberg boxes; Figure 3, left). Since a large and redundant basis set accompanies a high T-F resolution, adapting the transform framework to the signal properties and the needs of further signal analysis is one of the key steps. The wavelet transform (WT) provides a high-frequency resolution at low frequencies and a high-time resolution at high frequencies, thus making it a convenient and powerful tool for T-F analysis in biomedical applications.

**Figure 4:**
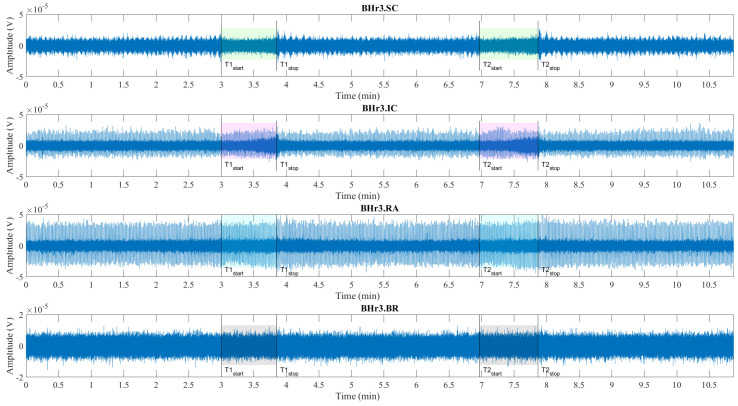
Time series of sEMG signals for three respiratory muscles EMG_SC_, EMG_IC_, and EMG_RA_ and one nonrespiratory muscle EMG_BR_, respectively, from top to bottom, which are given for a representative apneist **BHr3** (BHD Rank #1; Table 1). The first and second BH phases (BH1 and BH2) are marked with time markers (T1start/T1stop and T2start/T2stop, Figure 1) and colored rectangles, while their colors correspond to the sensor colors in Figure 2. In the final stages of BH, a gradual increase in the magnitudes of EMG_SC_, and especially EMG_IC_, was noticeable in both BH1 and BH2 periods.

**Figure 5:**
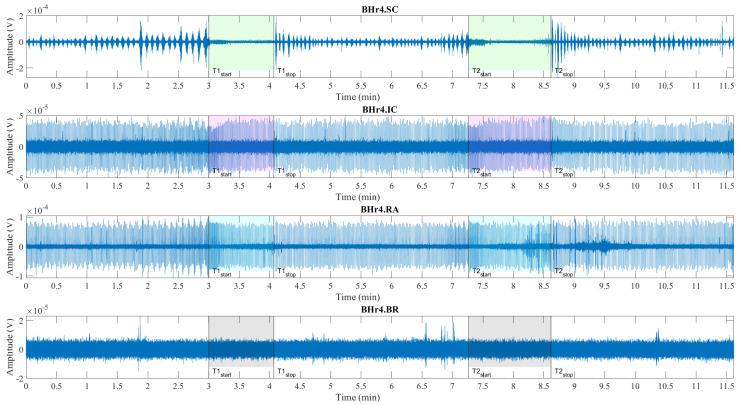
Time series of EMG_SC_, EMG_IC_, and EMG_RA_, and EMG_BR_, respectively, from top to bottom, which are given for a representative apneist **BHr4** (BHD Rank #1, highest BHD score; Table 1). Other descriptive notes in the caption for Figure 4. In contrast to BHr3, limited or no changes were observed in EMG_SC_ and EMG_IC_; however, abrupt changes in EMG_RA_ magnitudes were present in the final stages of BH2, indicating a different BH response mechanism.

The WT is a sparse signal decomposition using strictly local time–frequency waveform functions—the *wavelet family* (Appendix A). Depending on the construction of compactly supported wavelets, as well as the scaling and translating parameters, the WT provides a flexible time–frequency resolution—from high to critical resolution representation. This distinguishes two main classes of WTs, namely, the continuous wavelet transform (CWT; Appendix A.1), with high resolution and high redundancy due to the large overlap in the frequency and time domains, and the discrete wavelet transform (DWT; Appendix A.2) with a critical resolution and nonredundancy that is therefore easily invertible (for a comparison of CWT and DWT resolutions, see Figure 2, Figure 4, Figure 5 and Figure 6 in [37]). The orthogonal basis expansion methods provide a minimal set that can yield a sparse representation and, thus, a critical resolution, which, in the case of the WT, yields a perfect T-F plane tiling for the DWT (Figure 3, left). The concept of multiresolution analysis (MRA) combines continuous time wavelet theory and discrete filter banks [36]. The multiresolution theory of orthogonal wavelets can be implemented by a cascade of so-called conjugate mirror filters, which also yields a fast DWT (fDWT; Figure 3, right). The corresponding DWT, fDWT, and MRA are equivalent in decomposition and all provide the same expansion coefficients, but DWT-based MRA additionally provides the reconstructed signal components of the scales (frequency bands).

Regarding the advantages and disadvantages of CWT and DWT implementations, the general opinion is that CWT has excellent capabilities and performance in signal analysis, but not in signal synthesis due to its numerical instability, while the reverse is true for DWT. All this motivated the modifications of both WT methods (e.g., [37,38]) so that, in addition to CWT and DWT, several forms of redundant DWT have also been developed in order to optimally combine the advantages of both WTs. In this paper, we used the maximal overlap DWT (MODWT [38]; Section 3.2.1 and Appendix A.2) with good wavelet analysis and synthesis properties, in which translation and dilatation parameters correspond to integer sequences as in discretized CWT and dyadic scaling in DWT, respectively. The MODWT enables suitable analysis, because it does not average the frequencies contained in the signal, but the averaging takes place only in the time domain, thereby additionally stabilizing the signal by filtering (removing) high-frequency components that represent a form of noise.

Since most real-world signals are available as discrete time samples, and their processing is regularly implemented in a computational environment, as is the case here, the discretized WT versions were further considered.

### 3.1. Transient Localization and Scalograms

The CWT uses a near-continuous discretization (Equation (A6) in Appendix A.1) that transforms the time–domain waveform into the time–scale domain, where the scale can be converted to frequency, thus transforming the 1D-signal into the T-F plane. The absolute value of the CWT coefficients of the signal in this T-F plane provides a visual representation of the CWT, known as a scalogram. Similarly to the spectrogram, the scalogram can be interpreted as an energy density of the signal in the T-F plane. In this way, the scalogram offers a high-resolution and smoothly varying representation of the T-F domain, which enables high-fidelity signal analysis with the accurate localization of transients, the characterization of oscillatory behavior, etc. The CWT was used here to generate the scalograms for the visual detection of transient phenomena in respiratory muscle sEMG signals, as well as oscillatory cardiac (QRS) activity and their precise T-F localization. The part of the scalogram where edge effects became significant (COI; Appendix A.1) was mostly avoided and was located at the very margins of the signal by setting the lower frequency limit to 2 Hz, which also did not affect the BH1 and BH2 areas.

### 3.2. MODWT-Based Multiresolution Analysis

The MODWT is naturally defined for each signal sample, giving it significant advantages over DWT, including the possibility of its application to sequences of arbitrary length and translation invariance (overcoming the problem of effects attributable to the choice of a starting point for analysis in DWT). Since MODWT enables a reliable comparison of specific short-term signal intervals of interest, it was chosen as the appropriate WT technique for analyzing the dynamic patterns in the signals studied here.

In the MODWT method, a real-valued, uniformly sampled time series $x={\left\{x_{n}\right\}}_{n=1}^{N}$ is decomposed using a wavelet family:

(1) $$\psi_{j,k}\left[n\right]=\frac{1}{\sqrt{2^{j}}}\psi \left[\frac{n-k}{2^{j}}\right],$$

where j,k∈N index the scale/octave and the shift, respectively. By comparing the wavelet families in (1), (A6), and (A7), it follows that scales have the same octave range as in DWT, which means that there are no intermediate scales in the octave range as there are in CWT, as well as no downsampling by $2^{j}$ with scaling as in DWT.

The DWT (MODWT) of a function *x* is expressed by the following sum:

(2) $$X_{w}\left(j,k\right)=\sum_{j=1}^{J}\sum_{k=1}^{N}d_{j,k}\psi_{j,k}+\sum_{k=1}^{N}s_{J,k}\varphi_{J,k},$$

where, for each scale, *j*, $D_{j}={\left\{d_{j,k}\right\}}_{k=1}^{N}$, and $S_{J}={\left\{s_{J,k}\right\}}_{k=1}^{N}$ respectively represent the wavelet (*detail coefficients*) and the scaling (*smooth coefficients*) of the MODWT for corresponding mother (wavelet) functions $\Psi_{j}={\left\{\psi_{j,k}\right\}}_{k=1}^{N}$ and father (scaling) functions $\Phi_{J}={\left\{\varphi_{J,k}\right\}}_{k=1}^{N}$ (more details can be found in Appendix A.2, especially related to (A8)).

The standard MODWT algorithm implements circular convolution between the signal and the filters that must satisfy the conditions for an orthogonal wavelet. The MODWT decomposes the input signal *x*, sampled at a frequency $f_{s}$, into the J+1 coefficient sequences ${\left\{D_{j}\right\}}_{j=1}^{J}$ and $S_{J}$ within *J* logarithmically evenly spaced frequency intervals:

(3) $$\begin{array}{cc} \mathrm{scale}\;j\;\;(D_{j}): & \;\;[2^{-(j+1)}f_{s},2^{-j}f_{s}],\;\;\;j=1,2,\ldots ,J,\\ \mathrm{scale}\;J\;\;(S_{J}): & \;\;[0,2^{-(J+1)}f_{s}]. \end{array}$$

The MODWT is generally used to analyze the MODWT partitions of signal energy and variance.

#### 3.2.1. Wavelet Energy Spectrum (WES)

Unlike the DWT, which is an orthonormal WT that preserves signal energy (variance), the MODWT preserves energy (variance) by normalizing the MODWT filter energy to $2^{-j}$ at the *j*th level (instead of the DWT filters that have unit energy).

The discrete form of (A1) defines the MODWT partitions of the total signal energy at each decomposition level as the following:

(4) $${∥x∥}^{2}=E=\sum_{j=1}^{J}∥D_{j}{∥}^{2}+∥S_{j}{∥}^{2}=\sum_{j=1}^{J}E_{j}^{d}+E_{J}^{s}\neq \sum_{j=1}^{J}∥{\tilde{D}}_{j}{∥}^{2}+∥{\tilde{S}}_{J}{∥}^{2}.$$

The magnitude square of a wavelet coefficient, $d_{j,k}^{2}$, or a smooth coefficient, $s_{j,k}^{2}$, in (4) represent a local T-F energy density, and their sequence shows a time evolution of the energy density in a bandwidth corresponding to the scaling level *j* (compare (4) with (2) and (A8)). Their sums derived by individual frequency components, ${\left\{E_{j}^{d}\right\}}_{j=1}^{J}$ and $E_{J}^{s}$, characterize the signal energy distribution at different frequency subbands and represent a wavelet energy spectrum (WES).

The total normalized energy, obtained by dividing (4) by *E*, reflects the relative energy contribution

(5) $$\varepsilon_{j}=E_{j}/E$$

of each frequency interval to the total signal energy (this description is also known as the relative wavelet energy (RWE) in [39]). These normalized wavelet energy spectra $P^{\left(w\right)}\equiv \left\{{\left\{\varepsilon_{j}^{d}\right\}}_{j=1}^{J},\varepsilon_{J}^{s}\right\}$ of the respiratory signals EMG_SC_, EMG_IC_, and EMG_RA_ were used to analyze the correlation of the BH1 and BH2 maneuvers, as shown by scatter plots with marginal histograms in Section 4.2.3, as well as their changes during IBM in Section 4.2.4.

#### 3.2.2. Wavelet Variance

The wavelet variance provides a scale-based analysis of variance, i.e., it decomposes the variance of a time series into components related to different scales [40]. The variance is the mean squared difference from the mean, so the MODWT-based estimate of the time-independent wavelet variance is defined as follows:

(6) $$\sigma^{2}\left(x\right)=\bar{x^{2}}-{\bar{x}}^{2}=\frac{1}{N}\sum_{j=1}^{J}∥D_{j}{∥}^{2}+\frac{1}{N}∥S_{j}{∥}^{2}-{\bar{x}}^{2}=\sum_{j=1}^{J}\frac{E_{j}^{d}}{N}+\frac{E_{J}^{s}}{N}-{\bar{x}}^{2}.$$

If the signal has a mean value of zero, ${\bar{x}}^{2}=0$, the wavelet variance (6) is related to the scale-averaged WES of the signal *x*, i.e., it is related to the wavelet power spectrum (WPS). Given that the selection of the optimal number of decomposition levels *J* also implies the selection of a set of scales that significantly contributes to the signal variance; the variance of the *J*-level smooth (approximate) coefficients usually is not of interest, and the wavelet variance at scale *j* can be estimated by the variance of the *j*-level detail coefficients, where $\sigma_{j}^{2}\left(x\right)=\frac{1}{N}{∥D_{j}∥}^{2}$. Wavelet variance with a moving fixed-width window was used to analyze transient phenomena during IBM in Section 4.2.4.

#### 3.2.3. MRA Component Correlation

Linked to our long-term goal of a robust analysis method for detecting OSAHS and improving physical fitness tests, we would like to test the reproducibility of the physiological responses of respiratory muscles. We performed reproducibility and correlation analysis by comparing the normalized WES of the BH1 and BH2 phases depicted in Figure 1. If we establish the actual equivalence of physiological responses as measured by the normalized WES, we are able to combine BH1 and BH2 data for an increased statistical significance of our conclusions.

### 3.3. Research Objectives and Steps

In the forthcoming analysis, we used wavelet coefficients to describe the signals in the T-F domain by means of scalograms, as well as to define the (relative) energies of the signal MODWT components and derive metrics such as the WES and moving average variance. For this purpose, we used wavelets and related methodologies, which were in some cases correlated with the BHD, through the key steps of study as follows:

**Step 1.** Defininition of the frequency ranges and time intervals that are important for sEMG signal analysis using the occupied bandwidth (OBW) estimates of power spectral density (PSD) for signal decomposition within MODWT and MODWT-based MRA.

**Step 2.** Calculation of the (normalized) WES, i.e., the (relative) energies of the MODWT components for the total BH period, and its initial and final stages with their further use for quantitative analysis of the muscle response (related to Steps 4 and 5). Discovery of the correlation of the WESs classified according to different criteria (subject type, types of muscles, and MODWT components) in order to determine the reproducibility of the IBM phenomenon, thereby studying the response specificities of different muscle types.

**Step 3.** The study of the trends of muscle activity changes in different frequency ranges and corresponding to self-adaptive mechanisms in response to the BH in order to better understand the physiological response to the diving reflex. This was achieved by variance calculation using the moving window method of MRA components, which is a useful research tool for identifying important scales and for detecting energy/power inhomogeneities.

**Step 4.** Determination of the reproducibility of the time–frequency response of the respiratory muscles by correlating the energy components of the sEMG signals for all signal classifications through comparison of the BH1 and BH2 phases (related to the long-term goal).

**Step 5.** The better understanding of mechanisms that are included in apneic response for inspiratory and expiratory muscles, with the purpose of improving a general fitness test that includes tissue oxygenation assessment. We defined the muscle and the frequency range in which the largest energy change occurred globally by studying the energy change between the initial and final stages of the BH phases.

## 4. Results and Discussion

This section presents our research results, which are accompanied by a corresponding discussion. Although subjects with similar interindividual characteristics within each of the two groups were recruited for this study, they showed significant inter- and intraindividual variability in physiological muscle responses to the designed BH maneuver (Figure 1), thereby reducing the statistical consistency of the results. To draw valid conclusions from these highly variable data, we focused on understanding the correlation between the physiological responses and the physical fitness/training within individuals. Hence, the paper is based on a qualitative case study research with a discussion of the physiological implications and a presentation of quantitative indicators with statistical significance. Physiological implications were analyzed and interpreted mainly in the context of hypoxic and/or hypercapnic response, as well as the neuromuscular junction of muscle fiber subtypes.

The two main sets of results presented in this paper include the following: (1) a primary one related to the sEMG time–frequency features during involuntary breathing movements (IBMs) and (2) a secondary one related to breath-hold durations (BHDs). Both result sets were generally modulated by many factors. Beside individual factors such as sex, age, body size, chest shape, diaphragm position, thoracic blood volume, blood hemoglobin content, metabolic rate, obesity, disease etc., other common factors such as initial lung volume and posture have significant influence on sEMG and BHD results [41,42]. Considering that, in this study, we mainly limited the research objectives to the sEMG response of the respiratory muscles to hypoxic and/or hypercapnic stimuli, the mentioned common factors were chosen to minimize their effects on EMG activity. This was achieved by reducing additional unwanted elastic forces in the respiratory muscles and changes in lung volume, thus reducing unwanted stimulation of the respiratory center control. Namely, most breath-holding studies are based on breath holding from fully inflated lungs or total lung capacity (TLC), but such an initial lung volume generates tension in all respiratory muscles to overcome increased inspiratory resistance and pulmonary pressure. This produces the inflation reflex and also increases the impact of the mentioned individual factors [43]. Overcoming these factors can be achieved by the breath-holding onset during totally relaxed respiratory muscles, which is characteristic of the lung volume at the end of a normal exhalation, i.e., the functional residual capacity (FRC). In terms of posture, a supine position increases the pressure of abdominal contents on the diaphragm, thus preventing the diaphragm from being fully relaxed at end expiration and significantly reducing thoracic volumes, which thus affects the resting end-expiratory lung volume or FRC. Compared to the upright sitting position, the supine position provides the diminished elasticity of the ribcage and diaphragm, as well as the reduction of the rib cage compliance by 30% and the total static compliance of the respiratory system by 60% [41]. All the stated reasons led us to design the experiment with a BH maneuver during upright sitting. Additionally, the BH onset from FRC and the upright sitting posture both reduce pulmonary blood flow resistance and volume, thereby preventing or reducing unwanted receptor stimulation.

Furthermore, we also selected subjects who were physically active, either professionally or recreationally, and who had normal body weights and fat as indicated by the body-mass index (BMI), wherein they were in the range of 21.8–25.3 $\mathrm{kg}/m^{2}$ (Table 1). The biggest deviation in terms of population coherence lay in the fact that two subjects, BHr7 and BHr12, were in the post-COVID-19 period with six and four weeks after recovery, respectively. Given that the development of post-COVID-19 syndrome is associated with the persistence of some symptoms such as silent hypoxia and muscle weakness [44], we wanted to compare the potential influence of silent hypoxia on the respiratory muscle response to BH.

### 4.1. Breath-Hold Duration

All the subjects were classified into groups of professional and amateur/recreational athletes after successfully completing the experimental protocol. Their resulting BHDs during the first and second voluntary apnea BHD1 and BHD2 are given in Table 1 through individual values, as well as through mean values μ and standard deviations σ (both per group and for all participants in total).

In this study, the BHD was of secondary interest as a parameter that allowed us to better interpret the primary interest—the sEMG spectral characteristics. Population-wide BHD values fit a normal distribution with 43.5±15.8 s (μ±σ in seconds), and the values less than σ away from the mean included 8 out of 12 apneists, i.e., there were only 2 apneists (BHr4 and BHr6) with the highest and 2 apneists (BHr12 and BHr1) with the lowest BHDs outside the σ interval. However, by comparing the spectral characteristics of the sEMG signals, it became more reasonable to rank them into subgroups according to μ±σ/2 (more details in Section 4.2), which resulted in the following:

- BHD Rank #1—BHr4 (72.5 p/m), BHr6 (63.0 p/m), BHr5 (54.5 p/m), BHr3 (52.0 p/m);
- BHD Rank #2—BHr10 (48.5 a/m), BHr8 (47.5 a/m), BHr11 (42.5 a/m), BHr9 (35.5 a/m);
- BHD Rank #3—BHr2 (29.5 p/f), BHr7 (28.0 a/m), BHr1 (25.5 p/f), BHr12 (22.5 a/m).

The average of the BHD1 and BHD2 values in seconds is shown in parentheses, and the abbreviations indicate the following terms: p—professional, a—amateur, m—male, f—female.

### 4.2. Respiratory Muscle EMG Response

Illustrations of muscle the sEMG recordings during the BH maneuver are shown for two typical classes of response, which are represented by an apneists BHr3 (BHD Rank #1) in Figure 4 and BHr4 (BHD Rank #1) in Figure 5. The visual analysis of these sEMG time series reveals some characteristic signal patterns for different muscle types.

Consistent with the findings of other researchers, the periodic increase and decrease in the magnitude of the EMG_SC_ signal during three-minute periods of spontaneous breathing was associated with the changes in SC activity during repeated inhalation and exhalation, respectively, whereas this periodicity was absent during BH (see BHr3.SC in Figure 4 and BHr4.SC in Figure 5). The periodic activity of the EMG_SC_ signal was more pronounced for BHr4, while an additional increase before apnea indicated hyperventilation (it did not fit the design of the BH maneuver, but it was a mild hyperventilation that was not observed during the measurement), which was one of the factors that contributed to this subject having the highest BHD score. In addition, more intense hypoxia caused the resumption of breathing with an intense and sometimes abnormal pattern, which, all together, allowed us to use the EMG_SC_ signal to fine tune the time marker Tstop when possible, due to significant interindividual specificity in the SC response.

The low-frequency, high-amplitude peaks in the EMG_IC_ and EMG_RA_ signals (BHr3.IC & BHr3.RA in Figure 4 and BHr4.IC & BHr4.RA in Figure 5) during spontaneous breathing represent the R peaks of the cardiac QRS complex and were associated with the well-known general cardiac interference in respiratory EMG recordings.

In the preBH periods in BHr4, by comparing the envelopes of the EMG_SC_ and EMG_RA_ signals (and, to a lesser extent, the EMG_IC_ signal), as well as changes in the RR interval (time between successive heartbeats), a correlation was observed that corresponded to respiratory sinus arrhythmia—which is a normal change in heart rhythm during breathing.

In addition, the EMG_IC_ and EMG_RA_ signals of apneist BHr4 in the advanced stage of BH showed an increase in the RR interval that corresponded to the slowing of the heart rate. During the BH phases, especially in its final stages, the ECG readings were mixed with respiratory EMG responses, particularly for the EMG_RA_ signal in the BHD Rank #1 group. The impact of this signal mixing is additionally shown in Section 4.2.1, where it can especially be observed for the final BH stages of the EMG_RA_ signal that the distribution of the bandlimited power spectrum across apneists showed both increasing and decreasing power depending on the dominance of these two independent sources and their response to the BH. The separation of components from this linear mixture of signals of different origin was performed using CWT and MODWT in Section 4.2.2, Section 4.2.3 and Section 4.2.4 when possible.

The signal patterns related to cardiac activities are not observable in the EMG_SC_ and EMG_BR_ signals (BHr3.BR in Figure 4 and BHr4.BR in Figure 5), as they may have had some limited ECG interference.

The main observed effects of the prolonged BH for these two representative cases were different. For the BHr3, it consisted in a gradual increase of the sEMG magnitudes of the inspiratory muscle signals starting from the second half of the BH periods. This was noticeable for the EMG_SC_ signal, but it was significantly more pronounced for the EMG_IC_ signal. For the BHr4, we observed a somewhat opposite effect—the abrupt changes in the magnitudes of the expiratory muscle RA signal.

The observed difference in the response of the inspiratory and expiratory muscles to the BH in BHr3 and BHr4 led to our main hypothesis that this difference is caused by different mechanisms. In order to test this hypothesis, we correlated the relative energies of the wavelet MRA components with the BHDs, which will be given in more detail in Section 4.2.4 (especially in Figure 15). Other significant individual variations in the respiratory neuromuscular control that resulted in a different muscular response to the BH maneuver are analyzed and discussed in more detail in Section 4.2.2 and Section 4.2.4.

#### 4.2.1. Power Spectral Analysis

The sEMG signals in the time domain show us different types of responses to breath holding. In order to understand in more detail how the frequencies and power of the signal change over time, we compared the responses in the initial and final stages of the BHs. To study the changes in the frequency response, we calculated the occupied bandwidth (OBW), which gives a frequency range of the signal containing 99% of the total integrated power of its spectrum. The OBW is the frequency difference between points where the integrated power crosses 0.5% and 99.5% of the total power in the spectrum. To determine the OBW, we first estimated a periodogram power spectral density using a rectangular window and then integrated the estimate using the Riemann sum (midpoint rule). The OBW was calculated for a time interval of 3 s, while the locations of the time intervals were shifted by 2 s inwards from the BH phase boundaries, i.e., the BH initial stage was calculated from Tstart+2 to Tstart+5 (in seconds), while the BH final stage was calculated from Tstop-5 to Tstop-2 (in seconds).

**Figure 6:**
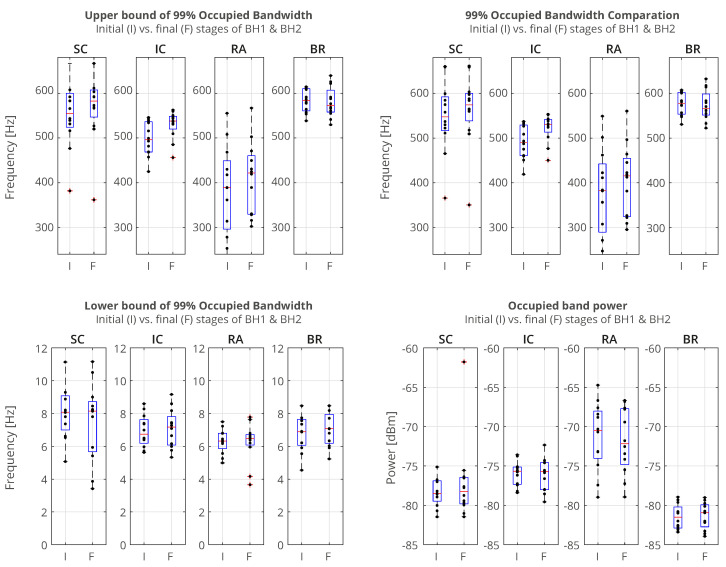
Comparison of the occupied bandwidth (OBW) estimates (the width of the frequency band that contains 99% of the power of the signal, the lower and upper bounds of the band, and the power in the band), of the muscle sEMG signals in the initial and final phases of breathholding (during BH1 and BH2). The lower bounds were about 7 Hz, which were a consequence of an AC-coupling filtering with 7 Hz highpass filter that strongly attenuated lower frequencies in the sEMG signals. As a result, the upper bounds were similar to the occupied bandwidth. The increase in occupied bandwidth was most pronounced for IC, followed by SC. The occupied band power was the highest for RA and the lowest for BR, which indicated that it was necessary to normalize the power in order to be able to compare the change in the initial and final BH phases for different muscles.

In Figure 6, we show a comparison of the occupied bandwidth (OBW) characteristics of the initial and final stages of the BHs (combined for BH1 and BH2) of the muscle sEMG signals for the case of the combined 12 naive apneists. Each column represents the OBW of the individual muscle sEMG signals of the SC, IC, RA, and BR muscles in order from left to right, which corresponds to the order of sensors from top to bottom in Figure 2. We can observe that the lower bounds approximately stayed the same, while the upper bounds increases for EMG_SC_, EMG_IC_, and EMG_RA_. As a result, the width of the OBW increased for these three signals, and it stayed the same for EMG_BR_. The occupied band power, which represents the signal power within the OBW, slightly increased for EMG_IC_, whereas it was not visibly changed for the other signals. We noticed that the combined effect of the heart rate drop took place, together with an increased response of the muscle sEMG signals, which was not easily discerned in these combined graphs. This motivated more detailed signal analysis of both the time and frequency so that we could better see various influences that overlapped each other and how the signals changed their frequencies and energies over time.

#### 4.2.2. Frequency Range of Muscle Response Using Scalograms

Our study used the Daubechies (*db*) wavelets family, specifically *db8* (of order eight), as one of the most suitable mother wavelets for the sEMG signal analysis according to [45]. In line with their recommendation [38], higher-order wavelets were used in order to achieve the lowest possible interactions between the decomposed components. The mother wavelets *db8* [45] and *sym8* [38] were compared; however, the *db8* wavelets slightly better concentrated the energy into active components, while their asymmetry did not affects the translation of the signal due to the high sampling frequency $f_{s}=1926$ Hz. Hence, our WMRA split the sEMG signals in the frequency bands D1–D8 and S8, as are defined in the following: D1 (481–963 Hz), D2 (241–481 Hz), D3 (120–241 Hz), D4 (60–120 Hz), D5 (30–60 Hz), D6 (15–30 Hz), D7 (7–15 Hz), D8 (4–7 Hz), and S8 (0–4 Hz).

**Figure 7:**
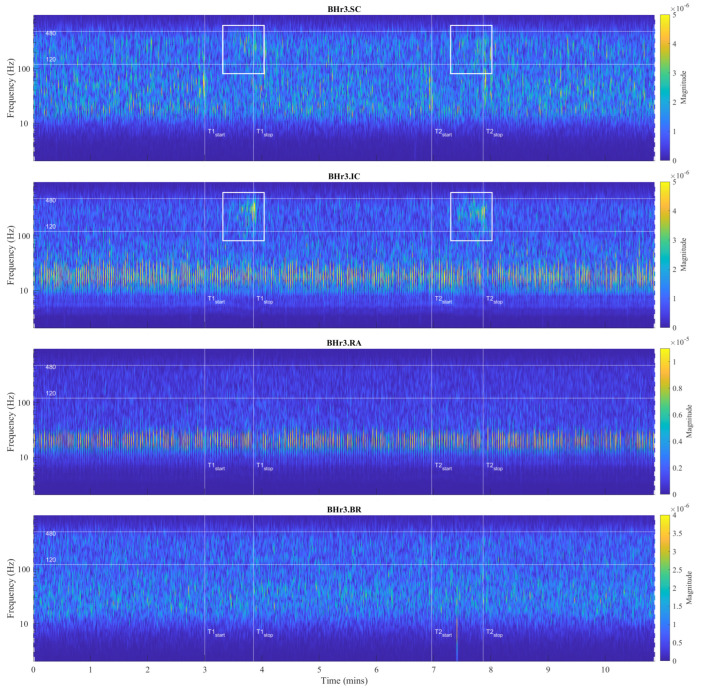
Scalograms of all four sEMG signals calculated for representative apneist **BHr3** (BHD Rank #1) (description of signals and time markers are the same as in Figure 4). Two specific frequency bands were observed: the first for EMG_IC_ signal and EMG_RA_ signal in the approximate frequency band (FB) range of 9–30 Hz, which normally corresponds to cardiac activity (the QRS wave), and the second for EMG_SC_ and EMG_IC_ signals in the approximate frequency band (FB) range of 120–480 Hz (D3—high FB (HFB) and D2—very high FB (VHFB)), which occurred in the final stage of BH and was related to the physiological breakpoint (see focused area indicated by white rectangular).

**Figure 8:**
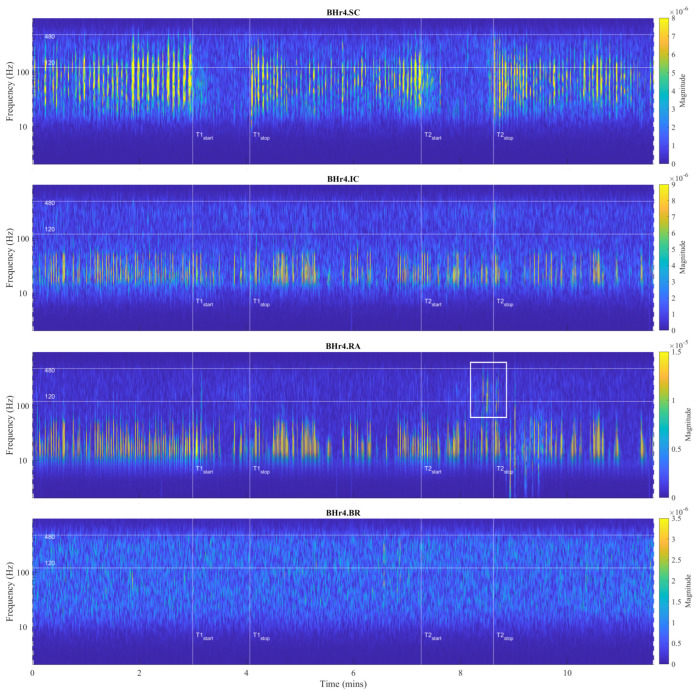
Scalograms of all four sEMG signals calculated for representative apneist **BHr4** (BHD Rank #1, highest BHD score) (description of signals and time markers are given in Figure 5). Similar to BHr3 (Figure 7), the same frequency range was observed for EMG_IC_ and EMG_RA_ signals related to cardiac activity. However, in contrast to BHr3, limited or no changes were observed in EMG_SC_ and EMG_IC_ signals; however, abrupt changes in EMG_RA_ signal in FB range of 80–300 Hz (predominantly in D4—medium FB (MFB) and D3—high FB (HFB)) during the final stages of BH2 (see focused area indicated by white rectangular), thus indicating a different BH response mechanism (compare with caption in Figure 5).

For the visual detection and the T-F localization of the transient phenomena in the respiratory muscle sEMG signals, as well as for the interfered oscillatory cardiac (QRS) activity, first we used scalograms (Section 3.1). CWT and MODWT analysis showed that, for the selected group of respiratory muscles in this study, the dominant muscle response and, thus, the physiological break point in all subjects, occurred in two respiratory muscles—either the inspiratory IC or the expiratory RA. The scalograms of the two representative cases for these two types of muscle responses are provided as follows:

- Figure 7 shows results for apneist BHr3 (BHD Rank #1) with a dominant response at the inspiratory IC (see sEMG time series for BHr3 in Figure 4);
- Figure 8 shows results for apneist BHr4 (BHD Rank #1, highest BHD score) with a dominant response at the expiratory RA (see sEMG time series for BHr4 in Figure 5).

In order to analyze the contributions of the higher and lower frequencies to the sEMG signals, it is worth noting that the sEMG frequency spectrum lies between 20 Hz (or lower) and 500 Hz and that, for respiratory muscles, the high frequency range is defined at 130–250 Hz, and the low frequency range is defined at 30–50 Hz [46]. In accordance with these standards, we assumed the following division of the frequency bands (FBs): very high FB—VHFB (250–500 Hz), high FB—HFB (130–250 Hz), medium FB—MFB (50–130 Hz), and low FB—LFB (30–50 Hz). By comparing the WMRA and standard FB division, we can determine the following approximate correlation: D2∼VHFB, D3∼HFB, D4∼MFB, and D5∼LFB.

For BHr3, the most pronounced muscle activity related to the apneic response was at the IC in the D2/VHFB and less in the D3/HFB, which was characterized by a progressive increase in the EMG_IC_ energy density centered around 300 Hz that started approximately in the middle of the BH and ended abruptly immediately after breath reinstatement. Very slight correlated changes in the same FBs were also observed for the EMG_SC_. In contrast, for the BHr4, no obvious increased sEMG activity was observed in the inspiratory muscles SC and IC, but there was abrupt activation of the expiratory muscle RA in the D3/HFB and less in the D4/MFB, which therefore resulted in a lower FB than for the inspiratory muscles.

Muscle activation accompanied by changes in the sEMG amplitude and energy/power spectral density is generally achieved by varying the combination of the motor unit (MU) recruitment and the modulation of the firing rate. The MU morphological features such as the number, spatial distributions (cross-sectional area), and the composition of muscle fibers are the major determinants of MU potential. Based on previous studies [47,48,49], these three partially dependent factors contribute differently to the variation in the motor unit action potential (MUAP) waveform and, thus, to the electromyographic activity and force generation—the geometric factors have a greater influence on the amplitude of the sEMG signal, while the fiber type has a greater influence on its spectral characteristics.

Three types of fibers are known to exist in human skeletal muscles, each with specific contractile and biochemical characteristics such that different compositions of fibers in a muscle are adapted to muscle functions in the body. The main biochemical characteristics of the fibers are related to the metabolic pathways for energy supply and oxidative capacity. According to the contractile properties and oxidative capacity, fibers are commonly categorized into slow-twitch slow oxidative (type 1), fast-twitch fast oxidative/glycolytic (2A), and fast-twitch fast glycolytic fibers (2X). Type 1 fibers use mainly oxidative phosphorylation, which makes them resistant to fatigue and, hence, are mainly involved in aerobic activities and endurance exercises. In contrast, type 2 fibers rely mainly on glycolytic metabolic pathways for energy supply, have faster contraction velocities than type 1 fibers, and are not fatigue-resistant. These types of fibers are predominant in the skeletal muscles of adult humans, but, in addition to them, these muscles of specific functions may contain other types, as well as “hybrid” types of fibers [50].

The recruitment pattern of MUs usually follows the principle of orderly recruitment according to their motor neuron size. Because MUs with slow type 1 fibers have smaller motor neurons, and those with fast-twitch type 2 fibers have larger ones, the order of recruitment is also related to MU fiber type composition. Hence, skeletal muscle contraction involves progressive recruitment in an additive manner and in the following order: type1→type2A→type2X [50]. Since an interference signal of all generated MUAPs defines the properties of the sEMG, the MU recruitment pattern reflects the spectral characteristics of the muscle activity such that fast fibers generate higher frequencies within the myoelectric spectrum than slow fibers [47]. The *hypoxia*-induced progressive activation of larger MUs with faster fibers during apnea can significantly explain the pattern of gradual changes in the EMG_SC_ and EMG_IC_ spectral characteristics in inspiratory muscles of BHr3 [9,51], but this explanation is unsatisfactory for the case of apneist BHr4 (Figure 8).

Although the generated MUAPs mainly depend on the MU recruitment pattern, other specific factors can significantly influence the modulation of the firing rate of MUAPs. In addition to hypercapnia induced by prolonged apnea, muscle contractions cause an increase in the concentration of lactic acid and other metabolic products, which leads to a decrease in intracellular pH and, consequently, to a decrease in muscle fiber conduction velocity [47,48,49]. The compensatory mechanism in maintaining or increasing the muscle contractile force despite the decrease in fiber conduction velocity induces a direct change in the shape of the MUAP waveform, which leads to an increase in various parameters of the waveform (duration, area, amplitude, etc.). Thus, these changes in MUAP characteristics induced by pH reduction directly influence greater electromyographic responses, but they do not imply correspondingly greater MU recruitment and muscle activation. In the case of the BHr4 apneist, as well as other apneists that held their breath for about 1 min at FRC (which at TLC could correspond to approximately twice as long the BHD [43]), we suggest that the sudden activation of the RA was primarily the result of *hypercapnic* stimulation.

The observed differences in the responses of the respiratory muscles to apnea-induced hypoxia and hypercapnia for apneists BHr3 and BHr4 are supported by well-known differences in the response of respiratory muscles to hyperventilation. Namely, for the equivalent minute volume, hypoxia mainly stimulates inspiratory muscles, while hypercapnia stimulates both inspiratory and expiratory groups [41]. Moreover, while diaphragmatic EMG activity increases in response to both hypercapnia and hypoxia, expiratory muscle activity increases almost exclusively during hypercapnic hyperventilation [42]. Abdominal muscles are generally expiratory, and RA, one of the important abdominal expiratory muscles, is known to play an important respiratory role during physical exercise and hypercapnia [42].

**Figure 9:**
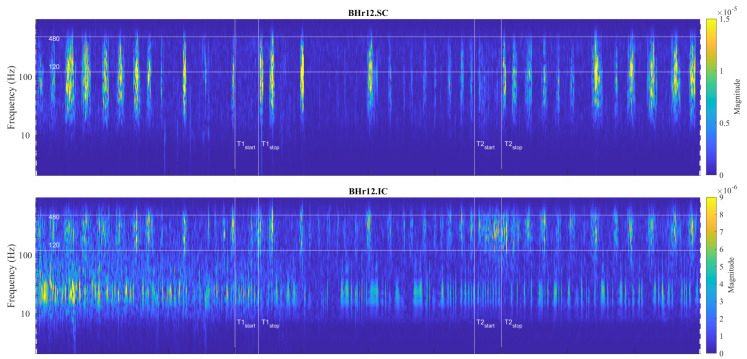
Correlations of neuromuscular coupling for inspiratory muscles SC and IC were demonstrated by comparing scalograms for EMG_SC_ and EMG_IC_ signals of naive apneist **BHr12** (BHD Rank #3). Correlation of areas of high energy density for EMG_SC_ and EMG_IC_ signals in the T-F plane were characteristic for the approximate FB of 120–480 Hz. This was the same FB for the increased energy density of the BH final stages for EMG_IC_ signals in both BHr12 and BHr3, as shown in Figure 7, thus indicating that this FB is characteristic of increased IC electromyographic activity, regardless of the primary stimulus.

It was thought that hypoxia and hypercapnia were simply additive in their effects, but, due to complexity, a hybrid model of integration of possible additive, hyperadditive, and hypoadditive interactions between the central and peripheral chemoreceptors in relation to systemic $P_{{\mathrm{CO}}_{2}}$ was proposed, where the form of interaction generally depends on the behavior and/or metabolism of the individual [52,53]. In this regard, the case of apneist BHr12 (BHD Rank #3, the lowest BHD score) is particularly indicative, where we can observe two phenomena by comparing the scalograms for the EMG_SC_ and EMG_IC_ signals shown in Figure 9. First, we observed a correlation of the neuromuscular activity for the SC and IC inspiratory muscles in the approximate frequency range of 120–480 Hz, which constituted the same characteristic D2/VHFB and D3/HFB results that were present in the IC and partially SC muscle responses to apnea in the BHr3 (scalograms were adopted to assess muscle cocontraction [54]). This correlation could be partly attributed to the intercostal–scalene reflex related to the phrenic nerve [55]. Second, there was a disproportionate increase in the IC electromyographic activity compared to the breath-hold duration (average BHD was 22 s, which was a period in which BHr3 and BHr4 had no response). The explanation may be in the mentioned ’silent’ or ’apathetic’ hypoxia and respiratory muscle weakness as the consequences of the previously acquired disease of COVID-19, while it is also known that COVID-19 can induce lactic acid production accompanied by uncompensated respiratory acidosis, decreased plasma pH, hypercapnia, and more [44]. This can be linked to the previous comment regarding a decrease in muscle fiber conduction velocity and an increase in sEMG activity without a proportional increase in muscle activity. Similarly, some other respiratory diseases can lead to a disproportionate increase in neural respiratory drive directed towards the respiratory muscles [48,49]. The correlation of the respiratory muscles with physical performance/training is also shown by respiratory muscle training [56,57].

**Figure 10:**
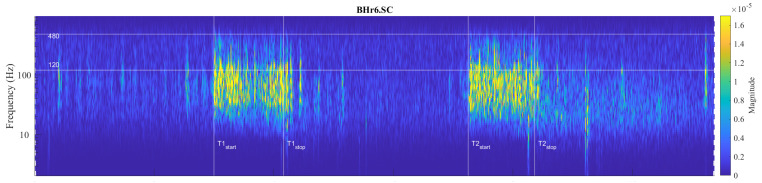
Individual specificities of neuromuscular coupling in participants for SC were demonstrated by comparing the scalograms for EMG_SC_ signals of apneist **BHr6** (BHD Rank #1) with BHr4 (BHD Rank #1, which are presented in Figure 8). During the BH maneuver, participant BHr4 (rower) showed higher SC activity during spontaneous breathing (areas of high energy density for EMG_SC_ signal in the T-F plane are related to inspiration), while BHr6 (diver) showed greater SC activity during breath holding.

Large interindividual variations in the physiological response to hypoxic and/or hypercapnic stimulation during apnea could be explained by a heterogeneous tissue of human skeletal muscle, as well as metabolic, energetic, and neuromuscular differences. Our analysis showed that, among the selected muscles, the individual specificities of the neuromuscular coupling in the participants were most pronounced in the SC, as demonstrated in the case of the apneist BHr6 (BHD Rank #1)—who is a professional diver (Figure 10).

The claims we made in this section could be partially confirmed if a relationship could be established between the spectral features of the sEMG activity and the BHD, so we first examined the reproducibility of the spectral features of the sEMG activity during the BH1 and BH2 using wavelet MRA (WMRA).

#### 4.2.3. Muscle Response Reproducibility Analyses Using WMRA

**Figure 11:**
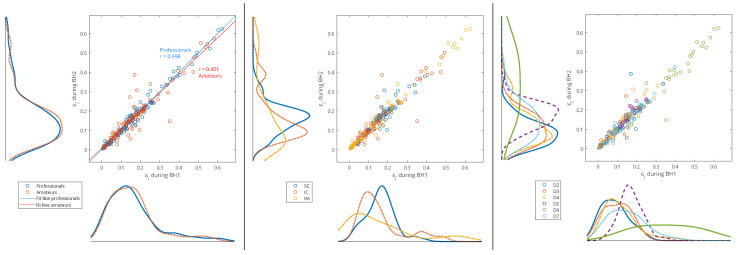
Joint distribution of relative energies of wavelet MRA (WMRA) components of respiratory signals EMG_SC_, EMG_IC_, and EMG_RA_ of all participants for three types of data grouping: professional/amateur (**left**), respiratory signal type (**middle**), and MRA components (**right**). The distributions were determined using a probability density estimate based on a normal kernel function. The lines of best fit of the two data sets, professionals and amateurs, are calculated using linear regression, where *r* is the appropriate estimate of the regression slope.

**Figure 12:**
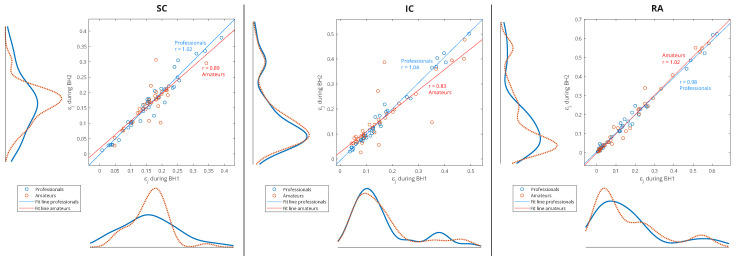
Separated distributions of relative energies of WMRA components of respiratory signals EMG_SC_, EMG_IC_, and EMG_RA_ of all participants for professional/amateur grouping. As in the caption of Figure 11, the lines of best fit of the two data sets, professionals and amateurs, are calculated using linear regression, where *r* is the appropriate estimate of the regression slope. A comparison of the distribution curves for professionals and amateurs shows that they most closely matched for the IC.

In order to analyze the relationship between the two groups of data—the distributions of the relative energies $\varepsilon_{j}$ of the WMRA components during BH1 and BH2, and, thus, muscle-activation reproducibility—the scatter plots with marginal histograms are presented in Figure 11 and Figure 12. They show the distributions of relative energies of the respiratory signals EMG_SC_, EMG_IC_, and EMG_RA_, which were determined using a probability density estimate based on the normal kernel function. To avoid a strong bias in the distributions, we excluded the D1, D8, and S8 scales, as these components had very small relative energies compared to the other scales and would otherwise create large peaks in the distribution around zero. In this way, we focused on the frequency components D2–D7 in the range from about 7 Hz to 500 Hz.

In Figure 11, the data of all the participants from both BH periods were used (*x*-axis: BH1 and *y*-axis: BH2), and the distributions are shown for three types of data grouping: professionals vs. amateurs (left), respiratory muscle signal type (middle), and WMRA components (right). We can notice that the energy distributions of professionals and amateurs were approximately the same (see Figure 11, left), which means that, on average, the populations of different subjects behaved similarly. At the same time, a strong correlation was observed between BH1 and BH2, since the coefficient of the linear regression model was approximately one, which provided the basis for the integral analysis of BH1 and BH2.

Observing the maximum values of probability density for the relative energies in Figure 11 (middle) indicates some biases between different muscles. The RA was characterized by a large number of components with low relative energy and a small number of components with a larger range of high relative energies, which mostly exceeded the relative energy values of the components for the SC and IC. On the contrary, the SC had the narrowest distribution, and the maximum shifted most to the right, which means that the relative energies by components were most evenly distributed (similar energies for all D2–D7 spectral components) and showed higher values compared to the RA and IC. The characteristics of the IC were somewhat in between the SC and RA, with two peaks of the accumulation of relative energies. Compared to Figure 11 (right), we can conclude that high relative energies mostly originated from the D6 signal component (15–30 Hz), which had an interference with cardiac electromyographic activity that pushed the energies towards higher values, especially for the EMG_RA_ and then for the EMG_IC_ signals (corresponding to the second distribution peak with a smaller number of components of higher relative energies). This general distribution of relative energy across the respiratory muscles is consistent with the energy density distribution in the T-F plane of their corresponding scalograms (Figure 7 and Figure 8).

Additionally, Figure 11 (right) shows that the relative energies at all scales had similar behavior, except for D5 and D6. While we gave an explanation for D6, the distribution of D5 most closely corresponded to the distribution of the SC, especially for high relative energies, which indicated the negligible influence of the D6 and D7 components. The narrow distribution of the D5 was a result of an accumulation of components of approximately similar relative energies that originated from different muscles and subjects—this indicates that it corresponded to a frequency range of the SC that was not significantly involved in apnea-induced muscle activity.

Since one of our goals was to find out which muscle was the most appropriate for IBM detection and characterization, we performed an analysis of the separate distributions of each of the respiratory muscles for grouping by sporting activity—professionals vs. amateurs (Figure 12). As there was also a partial correlation with the BHDs (Section 4.1) for this type of grouping, we gave an interpretation of some results in that context as well.

A consistent overlap of the distribution curves for professionals and amateurs was noticeable only for the IC, while for the RA and especially the SC, they differ (Figure 12). Since the IC distribution was very similar for widely varying values of the BHD (from ∼20 to ∼80 s), it follows that the apneic response of the IC is largely universal in the healthy population. In contrast, there was a significant difference in the distributions for the SC: the wide distribution for professionals points to a strong energy redistribution for a longer BH. Finally, for the RA muscle in the professional population, there was a distribution of energy from components with high relative energies to components with medium energies, which was not noticeable for amateurs (this can be more clearly seen in Figure 15, for Rank #1 subjects). This can be explained by the more pronounced heart rate drop that accompanied longer periods of BHs for professionals.

#### 4.2.4. Correlation Analyses of Muscle Activation and BH Duration Using WMRA

In Section 4.2.3, we analyzed the relative energies of the wavelet MRA components for the entire interval of the BH duration, but our goal now was to visualize and quantify the changes in relative energies during the development of the apneic response. We used the moving variance to visualize the transient process (as explained in Section 3.2.2), while the absolute difference of the normalized wavelet energy spectra (Section 3.2.1) between the BH final stage and BH initial stage was used to quantify energy changes during IBM.

**Figure 13:**
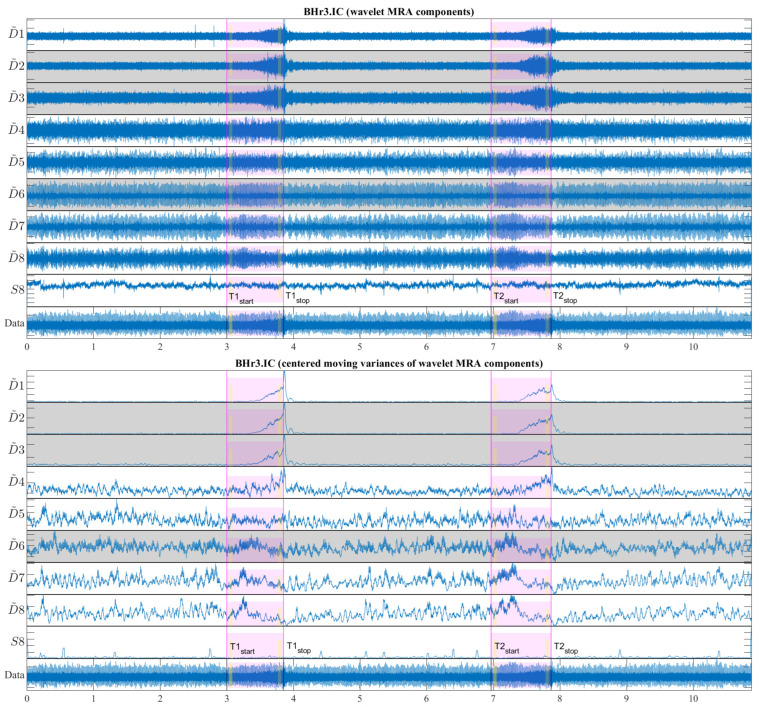
Wavelet MRA components of EMG_IC_ signal for **BHr3** (**above**) and their centered moving variances (**below**). The moving window had a time interval of 1.5 s—approximately 2 times the cardiac interbeat (RR) interval. The BH1 and BH2 phases are marked by rectangles in the magenta color chosen for the IC muscle, as marked in the Figure 4 and indicated in its caption. The narrow yellow rectangles indicate the BH initial stage and BH final stage that were used to quantify energy changes during the first involuntary breathing movement (IBM) shown in Figure 14 and Figure 15. Grayed-out areas correspond to the frequency bands with the highest relative energy changes for the IC muscle (compare with the boxplot representation of changes in relative energy across the scales related to IBM for the IC in Figure 14). The BHD of BHr3 was 52 s on average, while an energy increase started around the middle of the BH period and lasted until the end: in total 27 s.

The moving variance is a common tool for detecting short-term fluctuations and long-term trends or cycles in a time series. Since the moving variance is a type of convolution (nonlinear filter), it is calculated using the sliding window method. Here, it was applied to the WMRA components using a centered window of 1.5 s (2889 samples), where this fixed window length was chosen to contain approximately two cardiac RR intervals. Figure 13 shows a representative case of subject BHr3 (BHD Rank #1) for the IC muscle, where the upper part of the figure contains the WMRA components of the entire EMG_IC_ signal, and the lower part contains the corresponding filtered components using the centered moving variances.

From Figure 13, two synchronized processes are visible that occurred as a result of the apneic response (both started around the middle of BH1 and BH2): the first referred to a gradual increase in power for the high-frequency range D2/VHFB–D3/HFB (120–481 Hz) and to a lesser extent the D4/MFB, and the second referred to a gradual decrease in power for the frequency range D6–D7 (7–30 Hz) and to a lesser extent the D5/LFB.

In order to quantify the changes of relative energy across the scales related to the first IBM during the BHs, we compared the normalized wavelet energy spectra $P^{\left(\varepsilon \right)}\equiv \left\{{\left\{\varepsilon_{j}^{d}\right\}}_{j=1}^{J},\varepsilon_{J}^{s}\right\}$ (Section 3.2.1) at the BH initial stage, $P_{I}^{\left(\varepsilon \right)}$, and the BH final stage, $P_{F}^{\left(\varepsilon \right)}$. We calculated this change as the absolute difference between $P_{F}^{\left(\varepsilon \right)}$ and $P_{I}^{\left(\varepsilon \right)}$ such that, for each component, we obtained $△P^{\left(\varepsilon \right)}=P_{F}^{\left(\varepsilon \right)}$ − $P_{I}^{\left(\varepsilon \right)}$. The time intervals for calculating $△P^{\left(\varepsilon \right)}$ were 3 s with a shift of 2 s inwards from the BH phase boundaries, as was the case of the OBW in Figure 6 and as indicated in Figure 13 (the narrow yellow rectangles across the scales).

**Figure 14:**
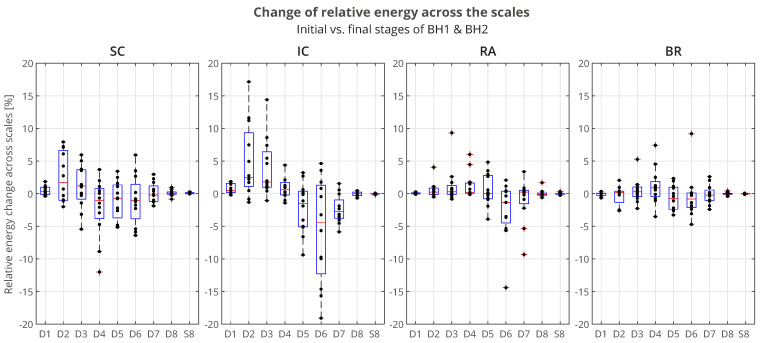
Boxplot representation of the changes in relative energy across the scales $△P^{\left(\varepsilon \right)}$ between the BH final and initial stages (as explained in the caption of Figure 13), calculated for the combined BH1 and BH2 periods. The changes were most pronounced for the IC muscle, in particular an increase of D2 and D3 components, with a synchronous decrease of the D6 component (compare with the captions of Figure 13 and Figure 15).

The distributions of $△P^{\left(\varepsilon \right)}$ of all the participants across the scales, grouped by muscles, is presented using boxplots in Figure 14. We can observe that the most pronounced increase in activity for the IC was primarily in the D2/VHFB component, followed by the D3/HFB, which were also the only increased components whose lower quartiles were above zero. It is also indicative that a similar trend was present in the same components of the SC such that the apneic response of the IC and SC was generally consistent in the frequency range above 120 Hz (D3 and D2 components). This correlated muscle activity of the inspiratory IC and SC muscles in the higher frequency range during apnea had been previously observed in the scalograms of the EMG_SC_ and EMG_IC_ signals for several individual apneists and was very apparent for apneist BHr12 (Rank #3; Figure 9), not only during apneic periods, but also during spontaneous breathing in the inspiratory phases.

At the same time, the D6 component of the IC had the largest energy drop similar to the D6 component of the RA, which could be attributed to heart rate drop (the SC and BR did not follow this trend due to the absence of limited electromyographic contributions from the cardiac signal).

For the BR muscle, a slight frequency redistribution of the muscle activity from a medium to high frequency band was also observed, especially a slight shift from the D5 to D4 frequency range. This can be explained by the limitation of blood flow to the limbs, not only as a consequence of the apneic response (sympathetically mediated peripheral vasoconstriction), but also of the metaboreflex (redistribution of blood flow to the respiratory muscles during their fatigue followed by a consequent restriction of blood flow to the locomotor muscles).

**Figure 15:**
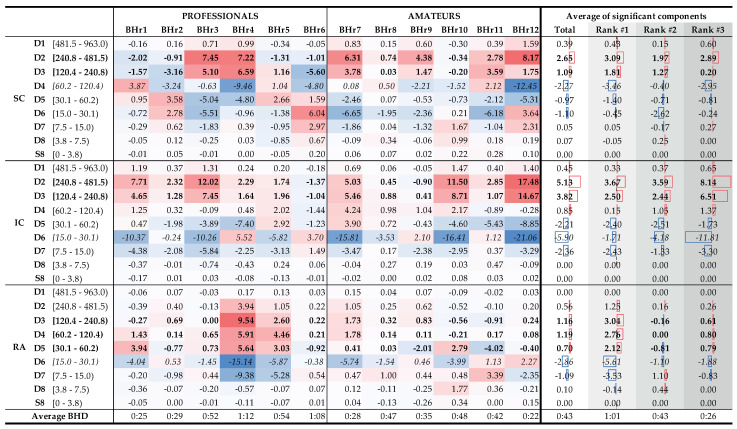
Changes in relative energy across the scales $△P^{\left(\varepsilon \right)}$ between the BH final and initial stages calculated for both BH1 and BH2 periods (**left**) and corresponding to the average values per scale for the total population and the population grouped by ranks (**right**). Scales with a significant positive change are displayed in **boldface**, and with negative change are *italic* (all values are expressed in percentages). Gradual highlighting from negative (blue) to positive (red) values was performed at the level of one muscle. In the part of the figure where the average values are calculated, the width of the red and blue boxes visualizes the size of the positive and negative change in values, respectively. Average values show the largest energy increase for D2 and D3 scales of EMG_IC_ and EMG_SC_ signals, regardless of the rank, as well as for D3, D4, and D5 scales of EMG_RA_ signal, but only for the Rank #1 group.

To understand which frequency bands had *significant* changes in energy in the SC, IC, and RA muscles, we calculated a change in relative energies across all the subjects and wavelet scales per muscle (see left side of Figure 15 that encompassed the 12 subjects labeled as professionals and amateurs). According to the central limit theorem, the distribution of values for each random process can be approximated with a Gaussian normal distribution for a large enough sample. As we were calculating a relative energy change for each person, which implied normalization, we obtained a process with a zero mean value for each person, but also for all the components of each muscle. Looking at Figure 15, in many cases, only a small relative change was observable, which could be attributed to a random process. In order to isolate the components with significant energy changes, we calculates a 95% confidence interval (CI), which, in this case, encompassed the majority of values where no significant change was observable. Values that were outside of this 95% CI were significantly different from the majority of the population and were the components of interest. On the right side of Figure 15, with the average change of significant components, we averaged, per rank, all the relative energies of a particular wavelet scale that were outside of the 95% CI, which thus represented components with significant change. For example, the value 3.09% for the D2 component of the Rank #1 subgroup represented an average value of significantly changed D2 components of the SC muscle for that subgroup.

The distributions of $△P^{\left(\varepsilon \right)}$ of the average values per scale for the total population and the BHD rank groups confirmed the insights and conclusions given for Figure 14 and also provided new ones in the context of the BHD.

According to the average values (Figure 15, columns Total, and Ranks #1, #2, and #3), the relative energy increase of the EMG_SC_ signal of the main two components the D2 and D3 calculated for the entire population was 88%, while, for the three BHD rank groups, the increased were 91%, 89%, and 78%, respectively. The corresponding average D2 and D3 values of the EMG_IC_ signal constituted 87% of the total energy increase, while, separated by the ranks, they were 93%, 81%, and 88%, respectively. The consistent contribution of the D2 and D3 components in the total energy increase of the EMG_SC_ and EMG_IC_ signals again indicates their correlated muscle activity in the higher frequency range, which we mentioned in the context of the results from Figure 9 and Figure 14. By comparing the joint percentage increases of these two components by BHD Ranks #1, #2, and #3 for the SC, which were 6.17%, 6.03%, and 14.65%, respectively, we conclude that the percentage increase was the most pronounced for the BHD Rank #3 group, especially with the BHr12 apneist, which could be related to a possible post-COVID hypoxic state.

From Figure 15, two sharp transitions of energy decreases and increases between adjacent components can be observed: first, between the D3 and D4 of the EMG_SC_ signal, which were visible for subjects with the BHD Ranks #1 and #3, and, second, between the D5 and D6 of the EMG_RA_ signal, which were visible only for subjects with the BHD Rank #1. It is particularly indicative that these most pronounced changes were correlated with the BHD rank groups: for the SC, it was for the apneists for whom hypoxia was more pronounced, either due to a significantly prolonged BH (BHD Rank #1) or due to the partial influence of possible chronic hypoxia and accompanied by uncompensated hypercapnia due to the post-COVID state (BHD Rank #3), while for the RA, it was for apneists for whom hypercapnia was more pronounced due to prolonged BH (BHD Rank #1). We consider that this phenomenon could be explained by MU recruitment: this would be due to a change in recruited MUs during the time evolution of the apneic response from smaller MUs of slow-twitch oxidative type 1 fibers to larger MUs of fast-twitch (oxidative) glycolytic type 2 fibers.

Figure 15 provides us with one of the most significant results that followed from the comparison of the distribution of energy changes for the EMG_RA_ signal in correlation with the BHD, where it is shown that the increase in activity in the D3–D5 components referred only to apnests with the first rank, more precisely, BHr4 and BHr5, whose BHDs lasted about 1 min. This observation is consistent with [42] according to which the RA responds almost exclusively to hypercapnia.

Finally, as an interesting and important fact, we can point out that most subjects from the Rank #2 group had a mild to moderate reaction to BH. Only subject BHr10, who had BHD2 (57 s) comparable to subjects with the BHD Rank #1, showed a more pronounced reaction on the IC muscle with the same characteristics (a pronounced increase of D2 and D3 components, with a synchronous decrease of the D6 component). A more detailed analysis in which we compared changes in component energies for BH1 and BH2 in subject BHr10 showed that the prolonging of BHD from 40 s (BHD1) to 57 s (BHD2) resulted in the greatest changes precisely in the mentioned components D2 and D3 (a total increase of 6%) and D6 (decrease 6%). This leads to the conclusion that longer breath holding, especially over 50 s, results in an increased activation of the IC muscle in healthy subjects.

The observed links between the sEMG activity and BHD, as well as their interpretation in the context of MU recruitment in relation to the respiratory muscle’s response to hypoxia/hypercapnia, are also supported by a recent study [58] in which an sEMG–NIRS correlation analysis was performed. Namely, using near-infrared spectroscopy (NIRS), as a standard method for measuring local tissue oxygenation, in correlation with sEMG activity of the locomotor muscle, a higher correlation between the EMG and NIRS signals was observed in participants with a more active lifestyle. It was also shown that subjects with the least active lifestyles had the lowest correlation. A reduced EMG–NIRS correlation indicates the influence of factors other than oxygenation, such as metabolic, energetic, and neuromuscular factors, which may be particularly pronounced in persons with a specific health condition, as in the case of BHr12 in the post-COVID state (more details in Section 4.2.2, Figure 9). All this gives us an additional argument that, by using the sEMG signals, we can evaluate oxygenation, even in cases when a disproportion between the sEMG activity and muscle response is observed.

Considering all these findings, we conclude that our setup allows for the assessment of skeletal muscle oxygen storage and current fitness levels: subjects with higher BHsD and a primarily hypercapnic response in the expiratory RA are more fit than subjects with shorter BHDs and primarily hypoxic response in the inspiratory IC.

This study has limitations that should be taken into account when validating the final conclusions related to the mentioned relationships between the spectral characteristics of respiratory muscles, their fiber activation, and metabolism during the apneic response. Future studies should include a bigger population and more respiratory muscles, especially the diaphragm, and they should use sEMG in combination with advanced techniques to assess blood flow and tissue oxygenation (NIRS) in order to obtain more accurate quantitative estimators of physical fitness.

## 5. Conclusions

Voluntary breath holding (apnea) serves as a model for studying various physiological concepts/principles and for testing various physical/clinical conditions. Breath holding and involuntary breathing movements during the ”struggle” phase of BH have been investigated extensively, but relatively few studies have been related to skeletal muscle characteristics in that context (e.g., muscle fiber type, fiber oxidative capacity, and fiber size). In this paper, we focused on the spectral characteristics of respiratory muscle electromyography (sEMG) signals during breath holding to find out the most appropriate muscle for IBM detection and characterization, as well as to assess the tissue hypoxia and/or hypercapnia response in healthy, physically active adults (professional and recreational athletes). The sEMG signals of three respiratory muscles—scalenus (SC), parasternal intercostal (IC), and rectus abdominis (RA)—as well as one locomotor muscle, brachioradialis (BR), were analyzed in the time–frequency (T-F) domain using the continuous and discrete wavelet transform (CWT and MODWT). Our investigation demonstrates that the dominant respiratory muscle response related to the physiological breakpoint (the first IBM) can be detected either in the inspiratory IC or the expiratory RA.

This pilot study preliminarily indicates a high reproducibility of physiological responses of respiratory muscles. We have observed that professional athletes had higher BHDs and a stronger hypercapnic response in the expiratory RA than recreational athletes, who had a stronger hypoxic response in the inspiratory IC. The hypoxic-sensitive inspiratory IC was activated faster and gradually in the frequency range of 250-450 Hz for recreational athletes (independent of the person and BHD). This is in contrast to the hypercapnic-sensitive expiratory RA, which was activated for professional athletes only at higher BHDs, but abruptly and in a person-specific range below 250 Hz (dependent of the person and BHD), which, in some individuals, may have partially overlapped with cardiac activity ranges. The comparison of the BHD and T-F features of the sEMG signals showed changes that were consistent with the mechanism of motor unit (MU) recruitment and the transition of slow-twitch oxidative (type 1) to fast-twitch (low oxidative) glycolytic (type 2) muscle fibers.

With this, we are able to assess skeletal muscle oxygen storage and see a potential to improve physical fitness tests by establishing a closer relationship between the physical condition with breath-hold duration (BHD) and the sEMG spectral characteristics of elevated myoelectric IC and RA activity during BH.

## Figures and Tables

**Table 1:** Sports-related, demographic, and anthropometric data of naive breath holders (BHr), together with their resulting breath-hold durations (BHDs) during the first and second voluntary apnea (BHD1 and BHD2). All data are labeled based on person belonging to professional and amateur groups according to sport discipline/activity and recorded as individual values, mean values, and standard deviations for both groups and for all participants in total.

	Sports Participation	Sport/Activity	Gender	Age (years)	Height (cm)	Weight (kg)	BMI ($\mathrm{kg}/m^{2}$)	BHD1 (min:s)	BHD2 (min:s)
BHr1	Professional	Swimming	Female	20.9	168	63	22.3	0:24	0:27
BHr2	Professional	Rowing	Female	29.4	180	75	23.2	0:31	0:28
BHr3	Professional	Rowing	Male	30.5	195	92	24.2	0:51	0:53
BHr4	Professional	Rowing	Male	26.9	197	90	23.2	1:04	1:21
BHr5	Professional	Athletics	Male	25.1	192	85	23.1	0:57	0:52
BHr6	Professional	Scuba diving	Male	27.2	186	80	23.1	1:10	1:06
	**PROFESSIONALS**		**MEAN**	**26.7**	**186.3**	**80.8**	**23.2**	**0:49**	**0:51**
			**SD**	**3.1**	**10.0**	**9.8**	**0.5**	**0:17**	**0:19**
BHr7	Amateur	Volleyball	Male	20.6	204	95	22.8	0:27	0:29
BHr8	Amateur	Jujutsu	Male	36.7	185	76	22.2	0:43	0:52
BHr9	Amateur	Jujutsu	Male	19.2	178	69	21.8	0:34	0:37
BHr10	Amateur	Jujutsu	Male	44.9	180	80	24.7	0:40	0:57
BHr11	Amateur	Jujutsu	Male	40.1	180	82	25.3	0:36	0:49
BHr12	Amateur	Yoga	Male	45.0	197	93	24.0	0:21	0:24
	**AMATEURS**		**MEAN**	**34.4**	**187.3**	**82.5**	**23.5**	**0:33**	**0:41**
			**SD**	**11.7**	**10.7**	**10.0**	**1.4**	**0:08**	**0:12**
	**TOTAL**		**MEAN**	**30.5**	**186.8**	**81.7**	**23.3**	**0:41**	**0:45**
			**SD**	**9.1**	**10.3**	**9.9**	**1.0**	**0:12**	**0:14**

## Data Availability

The data that support the findings of this study are available from the corresponding author, N.Ž.M., upon reasonable request.

## Abbreviations

The following abbreviations are used in this manuscript:

BH	Breath hold
BHD	Breath-hold duration
BR	Brachioradialis
CWT	Continuous wavelet transform
DWT	Discrete wavelet transform
FB	Frequency band
FRC	Functional residual capacity
IBM	Involuntary breathing movement
IC	Parasternal intercostal
LFB	Low-frequency band
LS	Least-square
MAP	Mean arterial pressure
MFB	Medium frequency band
MODWT	Maximal overlap discrete wavelet transform
MRA	Multiresolution analysis
MU	Motor unit
MUAP	Motor unit action potential
OBW	Occupied bandwidth
OSAHS	Obstructive sleep apnea–hypopnea syndrome
QRS	Ventricular depolarization and contraction (ventricular systole)
RA	Rectus abdominis
RMS	Root mean square
RWE	Relative wavelet energy
SC	Scalenus anterior at medium
sEMG	Surface electromyography
TLC	Total lung capacity
WES	Wavelet energy spectrum
WMRA	Wavelet multiresolution analysis
WPS	Wavelet power spectrum
WT	Wavelet transform

## Appendix A. Wavelet Transform (WT)

The WT theory is well known (one of the most comprehensive monographs on the subject to date is [36]), but here we present the basics for familiarity with our notations and a precise definition of the signal processing techniques we used.

### Appendix A.1. Continuous Wavelet Transform (CWT)

The WT decomposes a finite energy time signal $x\in L^{2}\left(R^{d}\right)$ into a linear combination of *dilated*/*scaled* and *translated* versions of a fixed prototype time function, called the *mother wavelet* $\psi \in L^{2}\left(R^{d}\right)$. For d=1, the CWT transforms a time domain waveform into a time–scale domain, where the scale can be converted to frequency, thus transforming the 1D-signal into the T-F plane.

Since signal *x* belongs to the space of square-integrable functions, $L^{2}\left(R\right)$, the square norm of the signal, also called the *energy* of the signal (the Parseval–Plancherel identity), is induced as an inner product:

(A1) $${∥x\left(t\right)∥}^{2}=\langle x,x\rangle =\int_{R}{\left|x\left(t\right)\right|}^{2}\;dt=\int_{R}x\left(t\right)\bar{x}\left(t\right)\;dt<\infty ,$$

where the overline represents an operation of the complex conjugate.

In the CWT, the mother wavelet dilated and translated versions yield the wavelet family, sometimes called the *child wavelets*.

(A2) $$\psi_{a,b}\left(t\right)=\frac{1}{\sqrt{a}}\psi \left(\frac{t-b}{a}\right),$$

where $a\in R_{+}$ defines dilation and also the scale, or the so-called octave band $\left[\frac{1}{a},\frac{2}{a}\right]$, while b∈R defines translation. The term $a^{-1/2}$ ensures that the norm of $\psi_{a,b}\left(t\right)$ is equal to one (a unit norm or unit energy). The wavelet family (A2) can be more compactly expressed using translation operator $T_{b}\psi \left(t\right)=\psi \left(t-b\right)$ and the dilation operator $D_{a}\psi \left(t\right)=a^{-1/2}\psi \left(t/a\right)$ such that the equivalence holds:

(A3) $$\psi_{a,b}\left(t\right)=T_{b}D_{a}\psi \left(t\right).$$

The CWT, analogous to (A1), is the inner product of the input signal x(t) and the wavelet family $\psi_{a,b}\left(t\right)$, which decomposes the signal x(t) into a series of the *wavelet coefficients*

(A4) $$X_{w}\left(a,b\right)=\langle x,\psi_{a,b}\rangle =\int_{R}x\left(t\right)\frac{1}{\sqrt{a}}\bar{\psi }\left(\frac{t-b}{a}\right)\;dt.$$

In the special case $x,h_{a}\in L^{1}\left(R\right)$ (continuous functions with compact support), the convolution can be written as an inner product, i.e., $\left(x*h_{a}\right)\left(t\right)=\langle x,T_{b}\tilde{h_{a}}\rangle$, where $\tilde{h_{a}}\left(t\right)=\bar{h_{a}}\left(-t\right)$ is the involution [59]. From (A3) and (A4), we obtain the following:

(A5) $$h_{a}\left(t\right)=D_{a}\bar{\psi }\left(-t\right)⟹X_{w}\left(a,b\right)=\left(x*h_{a}\right)\left(t\right),$$

i.e., the CWT can be interpreted as a continuous time *convolution* [59]. Since the filtering operation is also a convolution, the CWT is mathematically equivalent (A5) to passing the input signal x(t) through a series of wavelet filters $h_{a}\left(t\right)$ that are a reflected, conjugated, dilated, and normalized versions of the mother wavelet ψ(t).

Since the CWT does not have an analytical solution for most functions and can only be calculated numerically, and since most real-world signals are available as discrete time samples with their analytical forms being generally unavailable, a discretized version of the CWT is implemented in a computational environment. A discretized CWT, as with other types of WT, uses discretizing of the time axis by samples n∈Z and the scale axis in a dyadic (octave band) configuration (a=2) such that the discretized wavelet family has the following form:

(A6) $$\psi_{j,k}\left[n\right]=\frac{1}{\sqrt{2^{j/\nu }}}\psi \left[\frac{n-k}{2^{j/\nu }}\right],$$

where j,k∈Z index the scale/octave and the shift, respectively, and $\nu \in N_{>1}$ is the number of intermediated scales in an octave, which defines approximately the number of wavelet bandpass filters per octave (the filterbank). Using the filterbank generated by (A6), the CWT with the additional specification of the mother wavelet and the sampling frequency $f_{s}$, we return to the scale-to-frequency conversions. The absolute value of the CWT plotted as a function of time and frequency in a semilog graph represents the *scalogram*. The scalogram can be interpreted, similarly to the spectrogram, as an energy density of a signal in the T-F plane [59]. The part of the scalogram where edge effects become significant is defined as the cone of influence (COI), and it is the result of convolution near the signal boundaries, where it performs wavelet multiplication with undefined data outside the signal.

Although the CWT typically results in high-fidelity signal analysis, signal reconstruction from the coefficients (A4) using the inverse CWT is much less stable due to the high redundancy of the CWT (large shift and scale overlap).

### Appendix A.2. Discrete Wavelet Transform (DWT)

The discrete wavelet transform (DWT), as with the CWT, can be applied to a continuoustime signal or a discrete time signal (a real-valued uniformly sampled time series). The discreteness of the DWT is based on discrete shift and scale parameters that ensure signal reconstruction with minimum redundancy and loss of information (critical resolution representation). Since the shift and scale parameters are correlated, the DWT is generated on a fine-tuned time–scale lattice (instead of a time–scale plane as for the CWT).

In a special case, a signal description by the minimal number of wavelet coefficients possible can be obtained when the scaled and shifted wavelets form a multiresolution analysis (MRA), i.e., when the (mother) wavelet functions $\left\{\psi_{j,k}\left[n\right]:j,k\in Z\right\}$ and the newly introduced (*father*) scaling functions $\left\{\varphi_{j,k}\left[n\right]:j,k\in Z\right\}$ form an orthonormal basis of $L^{2}\left(R\right)$, so they are both orthogonal and normalized. This applies when the mother and father functions have a similar form with the same shift and dilate/scale parameters, while the mother functions are as in (A6) for ν=1 and $k⟼k2^{j}$:

(A7) $$\psi_{j,k}\left[n\right]=\frac{1}{\sqrt{2^{j}}}\psi \left[\frac{n-k2^{j}}{2^{j}}\right],$$

which means that there are no intermediate scales in the octave range, and there is a downsampling by $2^{j}$ with scaling.

However, a redundant representation offers a higher-fidelity signal analysis, as with the time (or shift) invariant and highly redundant DWT with no downsampling, which is called the maximal overlap discrete wavelet transform (MODWT) [38]. The MODWT uses values removed from the DWT by downsampling and has a form as in (A6) for ν=1, so the mother functions are rescaled or dilated by powers of two, as in the DWT, and translated by integers, as in the CWT, and, thus, it is defined for all samples sizes (any signal length).

Similar to the DWT, it can perform MRA, where a MODWT-based MRA is more reliable and provides a high-accuracy reconstruction of the discrete time signal $x={\left\{x_{n}\right\}}_{n=1}^{N}$ as follows:

(A8) $$x=\sum_{j=1}^{J}\sum_{k=1}^{N}d_{j,k}{\tilde{\psi }}_{j,k}+\sum_{k=1}^{N}s_{J,k}{\tilde{\varphi }}_{J,k}=\sum_{j=1}^{J}{\tilde{D}}_{j}+{\tilde{S}}_{J},$$

where $d_{j,k}=\langle x,\psi_{j,k}\rangle$ are the wavelet or *detail coefficients*, and $s_{j,k}=\langle x,\varphi_{j,k}\rangle$ are the scaling or *smooth coefficients* of the MODWT, while $\tilde{\psi }$ and $\tilde{\varphi }$ are the dual functions of ψ and φ, respectively. Orthogonal projections of the detail coefficients at scale/level *j* yield a sequence ${\tilde{D}}_{j}$ that defines the *j*th level MODWT-based MRA *signal detail*, and, analogously, ${\tilde{S}}_{J}$ is the *J*th level *signal smooth* (approximation). Adding the *j*th level signal detail to the *j*th level signal smooth gives the (j−1)th signal smooth, i.e., the signal approximation at an increased resolution. The relation (A8) also represents the inverse MODWT (iMODWT) and differs from the iDWT by the summation’s upper limit for *k*, where $N⟼N/2^{j}$ is derived due to downsampling in the DWT signal decomposition. Hence, for the DWT, the lowest resolution *J* is bounded by $2^{J}≯N$; this is in contrast to the MODWT pyramidal algorithm, which always yields *N* scaling coefficients for the next level and, thus, *J* is unbounded. The MODWT is generally used to analyze the MODWT partitions of signal energy and variance.

A time redundancy of the MODWT enables the accurate time alignment of the original signal features and the features of its decomposition coefficients $d_{j,k}^{2}$ and $s_{j,k}^{2}$ at each level, but it induces a certain degree of correlation among these coefficients. As a consequence, although the MODWT-based MRA components ${\tilde{D}}_{j}$ and ${\tilde{S}}_{j}$ accurately reveal the content of the original signal within the frequency bands, they do not preserve energy, as indicated by Equation (4). Using wider filters, i.e., those with a larger number of vanishing moments, can result in an approximate uncorrelatedness between wavelet coefficients across scales for certain time series [38].

Using filters, the DWT/MODWT results in a recursive relationship between the filter coefficients and the scaling and wavelet functions: the *low*pass halfband filter determine the scaling functions and produces $s_{j,k}$, while the *high*pass halfband filter determine the wavelet functions and produces $d_{j,k}$. Dyadic signal decomposition (analysis) and perfect reconstruction (synthesis) are fulfilled when two pairs of filters satisfy the conjugate quadrature filter (CQF) relationship, which means that the corresponding lowpass and highpass filters are orthogonal, while the corresponding analysis and synthesis filters are time-reversed.

## References

[1] Parkes M.J. (2006). Breath-holding and its breakpoint. Exp. Physiol..

[2] Lindholm P., Lundgren C.E. (2009). The physiology and pathophysiology of human breath-hold diving. J. Appl. Physiol..

[3] Bain A.R., Ainslie P.N., Barak O.F., Hoiland R.L., Drvis I., Mijacika T., Bailey D.M., Santoro A., DeMasi D.K., Dujic Z. (2017). Hypercapnia is essential to reduce the cerebral oxidative metabolism during extreme apnea in humans. J. Cereb. Blood Flow Metab..

[4] Cooper H.E., Parkes M.J., Clutton-Brock T.H. (2003). CO2-dependent components of sinus arrhythmia from the start of breath holding in humans. Am. J. Physiol.-Heart Circ. Physiol..

[5] Foster G.E., Sheel A.W. (2005). The human diving response, its function, and its control. Scand. J. Med. Sci. Sport..

[6] Dujic Z., Breskovic T. (2012). Impact of breath holding on cardiovascular respiratory and cerebrovascular health. Sport. Med..

[7] Skow R.J., Day T.A., Fuller J.E., Bruce C.D., Steinback C.D. (2015). The ins and outs of breath holding: Simple demonstrations of complex respiratory physiology. Adv. Physiol. Educ..

[8] Bain A.R., Drvis I., Dujic Z., MacLeod D.B., Ainslie P.N. (2018). Physiology of static breath holding in elite apneists. Exp. Physiol..

[9] Elia A., Gennser M., Harlow P.S., Lees M.J. (2021). Physiology, pathophysiology and (mal)adaptations to chronic apnoeic training: A state-of-the-art review. Eur. J. Appl. Physiol..

[10] Jerath R., Crawford M.W., Barnes V.A., Harden K. (2015). Widespread depolarization during expiration: A source of respiratory drive?. Med. Hypotheses.

[11] Palada I., Bakovic D., Valic Z., Obad A., Ivancev V., Eterovic D., Shoemaker J.K., Dujic Z. (2008). Restoration of hemodynamics in apnea struggle phase in association with involuntary breathing movements. Respir. Physiol. Neurobiol..

[12] Willie C.K., Ainslie P.N., Drvis I., MacLeod D.B., Bain A.R., Madden D., Maslov P.Z., Dujic Z. (2015). Regulation of Brain Blood Flow and Oxygen Delivery in Elite Breath-Hold Divers. J. Cereb. Blood Flow Metab..

[13] Cross T.J., Kavanagh J.J., Breskovic T., Zubin Maslov P., Lojpur M., Johnson B.D., Dujic Z. (2013). The Effects of Involuntary Respiratory Contractions on Cerebral Blood Flow during Maximal Apnoea in Trained Divers. PLoS ONE.

[14] Joulia F., Steinberg J.G., Faucher M., Jamin T., Ulmer C., Kipson N., Jammes Y. (2003). Breath-hold training of humans reduces oxidative stress and blood acidosis after static and dynamic apnea. Respir. Physiol. Neurobiol..

[15] Trembach N., Zabolotskikh I. (2017). Breath-holding test in evaluation of peripheral chemoreflex sensitivity in healthy subjects. Respir. Physiol. Neurobiol..

[16] Trembach N., Zabolotskikh I. (2017). The influence of age on interaction between breath-holding test and single-breath carbon dioxide test. BioMed Res. Int..

[17] Barnai M., Laki I., Gyurkovits K., Angyan L., Horvath G. (2005). Relationship between breath-hold time and physical performance in patients with cystic fibrosis. Eur. J. Appl. Physiol..

[18] Yeo J., Kim J.Y., Kim M.H., Park J.W., Park J.K., Lee E.B. (2022). Utility of the breath-holding test in patients with systemic sclerosis. Rheumatology.

[19] Li Y., Wang Y. (2021). Obstructive Sleep Apnea-hypopnea Syndrome as a Novel Potential Risk for Aging. Aging Dis..

[20] Drager L.F., Togeiro S.M., Polotsky V.Y., Lorenzi-Filho G. (2013). Obstructive Sleep Apnea: A Cardiometabolic Risk in Obesity and the Metabolic Syndrome. J. Am. Coll. Cardiol..

[21] Athayde R.A.B., Oliveira Filho J.R.B., Lorenzi Filho G., Genta P.R. (2018). Obesity hypoventilation syndrome: A current review. J. Bras. Pneumol..

[22] Messineo L., Taranto-Montemurro L., Azarbarzin A., Oliveira Marques M.D., Calianese N., White D.P., Wellman A., Sands S.A. (2018). Breath-holding as a means to estimate the loop gain contribution to obstructive sleep apnoea. J. Physiol..

[23] Stewart M., Bain A.R. (2021). Assessment of respiratory effort with EMG extracted from ECG recordings during prolonged breath holds: Insights into obstructive apnea and extreme physiology. Physiol. Rep..

[24] Ostojić M., Milosavljević M., Kovačević A., Stokić M., Stefanović D., Mandić-Gajić G., Jeličić L. (2020). Changes in power of surface electromyogram during breath-holding. Srp. Arh. Za Celok. Lek..

[25] Konrad P. (2005). The ABC of EMG: A Practical Introduction to Kinesiological Electromyography.

[26] Ginszt M., Zieliński G. (2021). Novel Functional Indices of Masticatory Muscle Activity. J. Clin. Med..

[27] Medved V., Medved S., Kovač I. (2020). Critical Appraisal of Surface Electromyography (sEMG) as a Taught Subject and Clinical Tool in Medicine and Kinesiology. Front. Neurol..

[28] Guerra I., Aidar F.J., Greco G., de Almeida-Neto P.F., De Candia M., de Araújo Tinoco Cabral B.G., Poli L., Filho M.M., Carvutto R., Silva A.F. (2022). Are sEMG, Velocity and Power Influenced by Athletes’ Fixation in Paralympic Powerlifting?. Int. J. Environ. Res. Public Health.

[29] Fidalgo-Herrera A., Miangolarra-Page J., Carratalá-Tejada M. (2021). Electromyographic traces of motor unit synchronization of fatigued lower limb muscles during gait. Hum. Mov. Sci..

[30] Barroso-García V., Gutiérrez-Tobal G.C., Gozal D., Vaquerizo-Villar F., Álvarez D., del Campo F., Kheirandish-Gozal L., Hornero R. (2021). Wavelet Analysis of Overnight Airflow to Detect Obstructive Sleep Apnea in Children. Sensors.

[31] Perini R., Tironi A., Gheza A., Butti F., Moia C., Ferretti G. (2008). Heart rate and blood pressure time courses during prolonged dry apnoea in breath-hold divers. Eur. J. Appl. Physiol..

[32] Hermens H.J., Freriks B., Disselhorst-Klug C., Rau G. (2000). Development of recommendations for SEMG sensors and sensor placement procedures. J. Electromyogr. Kinesiol..

[33] Merletti R., Muceli S. (2019). Tutorial. Surface EMG detection in space and time: Best practices. J. Electromyogr. Kinesiol..

[34] De Luca C.J., Kuznetsov M., Gilmore L.D., Roy S.H. (2012). Inter-electrode spacing of surface EMG sensors: Reduction of crosstalk contamination during voluntary contractions. J. Biomech..

[35] Boyer M., Bouyer L., Roy J.S., Campeau-Lecours A. (2023). Reducing Noise, Artifacts and Interference in Single-Channel EMG Signals: A Review. Sensors.

[36] Mallat S. (2009). A Wavelet Tour of Signal Processing: The Sparse Way.

[37] Arts L.P.A., van den Broek E.L. (2022). The fast continuous wavelet transformation (fCWT) for real-time, high-quality, noise-resistant time–frequency analysis. Nat. Comput. Sci..

[38] Percival D.B., Walden A.T. (2000). The Maximal Overlap Discrete Wavelet Transform. Wavelet Methods for Time Series Analysis.

[39] Rosso O., Martin M., Figliola A., Keller K., Plastino A. (2006). EEG analysis using wavelet-based information tools. J. Neurosci. Methods.

[40] Percival D.B., Walden A.T. (2000). The Wavelet Variance. Wavelet Methods for Time Series Analysis.

[41] Lumb A.B., Lumb A.B. (2017). Chapter 2—Elastic Forces and Lung Volumes. Nunn’s Applied Respiratory Physiology.

[42] Lumb A.B., Lumb A.B. (2017). Chapter 5—Pulmonary Ventilation. Nunn’s Applied Respiratory Physiology.

[43] McCulloch P.F., Gebhart B.W., Schroer J.A. (2021). Large Lung Volumes Delay the Onset of the Physiological Breaking Point During Simulated Diving. Front. Physiol..

[44] Rahman A., Tabassum T., Araf Y., Al Nahid A., Ullah M.A., Hosen M.J. (2021). Silent hypoxia in COVID-19: Pathomechanism and possible management strategy. Mol. Biol. Rep..

[45] Kilby J., Gholam Hosseini H. Wavelet analysis of surface electromyography signals. Proceedings of the The 26th Annual International Conference of the IEEE Engineering in Medicine and Biology Society.

[46] (2002). ATS/ERS Statement on Respiratory Muscle Testing. Am. J. Respir. Crit. Care Med..

[47] Wakeling J.M., Uehli K., Rozitis A.I. (2006). Muscle fibre recruitment can respond to the mechanics of the muscle contraction. J. R. Soc. Interface.

[48] Duiverman M., de Boer E., van Eykern L., de Greef M., Jansen D., Wempe J., Kerstjens H., Wijkstra P. (2009). Respiratory muscle activity and dyspnea during exercise in chronic obstructive pulmonary disease. Respir. Physiol. Neurobiol..

[49] Cavalcanti J.D., Fregonezi G.A.F., Sarmento A.J., Bezerra T., Gualdi L.P., Pennati F., Aliverti A., Resqueti V.R. (2022). Electrical activity and fatigue of respiratory and locomotor muscles in obstructive respiratory diseases during field walking test. PLoS ONE.

[50] Schiaffino S., Reggiani C. (2011). Fiber Types in Mammalian Skeletal Muscles. Physiol. Rev..

[51] Elia A., Wilson O.J., Lees M., Parker P.J., Barlow M.J., Cocks M., O’Hara J.P. (2019). Skeletal muscle, haematological and splenic volume characteristics of elite breath-hold divers. Eur. J. Appl. Physiol..

[52] Wilson R.J.A., Day T.A. (2013). CrossTalk opposing view: Peripheral and central chemoreceptors have hypoadditive effects on respiratory motor output. J. Physiol..

[53] Teppema L.J., Smith C.A. (2013). CrossTalk opposing view: Peripheral and central chemoreceptors have hyperadditive effects on respiratory motor control. J. Physiol..

[54] Di Nardo F., Morano M., Strazza A., Fioretti S. (2022). Muscle Co-Contraction Detection in the Time-Frequency Domain. Sensors.

[55] Butler J.E., McKenzie D.K., Gandevia S.C. (2003). Reflex inhibition of human inspiratory muscles in response to contralateral phrenic nerve stimulation. Respir. Physiol. Neurobiol..

[56] Cirino C., Marostegan A.B., Hartz C.S., Moreno M.A., Gobatto C.A., Manchado-Gobatto F.B. (2023). Effects of Inspiratory Muscle Warm-Up on Physical Exercise: A Systematic Review. Biology.

[57] Manchado-Gobatto F.B., Torres R.S., Marostegan A.B., Rasteiro F.M., Hartz C.S., Moreno M.A., Pinto A.S., Gobatto C.A. (2022). Complex Network Model Reveals the Impact of Inspiratory Muscle Pre-Activation on Interactions among Physiological Responses and Muscle Oxygenation during Running and Passive Recovery. Biology.

[58] Daniel N., Sybilski K., Kaczmarek W., Siemiaszko D., Małachowski J. (2023). Relationship between EMG and fNIRS during Dynamic Movements. Sensors.

[59] Gröchenig K. (2001). Basic Fourier Analysis. Foundations of Time-Frequency Analysis.

